# Supplementary material for: Stenus-inspired, swift, and agile untethered insect-scale soft propulsors
Source: Nat Commun. 2024 Feb 19;15:1491. doi: 10.1038/s41467-024-45997-3 (PMC10876683; doi:10.1038/s41467-024-45997-3)
Supplement: Supplementary file 1 — Supplementary Information [file 41467_2024_45997_MOESM1_ESM.pdf]

Supplementary Information

Stenus-inspired, swift and agile untethered insect-scale soft propulsors

Xingxing Ke[1], Haochen Yong[1], Fukang Xu[1], Han Ding[1], Zhigang Wu[1]*

[1] State Key Laboratory of Intelligent Manufacturing Equipment and Technology, Huazhong University of Science and Technology, Wuhan 430074, China

*Corresponding author. Email: zgwu@hust.edu.cn

This PDF file includes:

Supplementary Notes
Supplementary Figures
Supplementary Tables
Supplementary Methods
Supplementary References

Supplementary Notes

Supplementary Note 1. Self-decoupling manipulation of Uni-SoPros

Real-time control of direction and propulsion is critical for the agile maneuverability of soft propulsors in dynamic environments. Herein, we utilize magnetic fields to serve as a remote trigger to manipulate the states of Uni-SoPros. To simultaneously achieve the decoupled control of direction and propulsion, we codesigned the swerving and propulsion trigger mechanisms at the same time in our Uni-SoPros. Specifically, a 3D magnetic field **B** is precisely generated by a Helmholtz coil, and the magnetic field **B** is decomposed to a horizontal component $\mathbf{B}_{\text{x-y}}$ and a vertical component $\mathbf{B}_{\text{z}}$. The component magnetic field $\mathbf{B}_{\text{x-y}}$ is used for real-time direction control and the component magnetic field $\mathbf{B}_{\text{z}}$ is used for real-time propulsion control. Based on this strategy, the orientation and locomotion state can be simultaneously manipulated wholly via a programmable time-varying 3D magnetic field **B** merely.

For real-time propulsion control, a magnetic responsive tail inspired by rove beetles' abdomen is introduced for surfactant release and then propulsion control as rove beetle does. The surfactant can be locally delivered onto the water surface when the magnetic tail is deflected down under the trigger of the magnetic field component $\mathbf{B}_{\text{z}}$. Consequently, the surfactant released onto water surface can cause a gradient surface tension and hence produce a Marangoni propulsion. Moreover, the fuel delivery can be fast terminated by an upward deflection of the magnetic tail. Therefore, the magnetic field component $\mathbf{B}_{\text{z}}$ directly determines the propulsion states of Uni-SoPros.

Here, magnetic torques (*1*) on the magnetic tail can be expressed as follows,

$$\vec{\tau}_{\mathrm{d}} = \int_{v_{\mathrm{mt}}} \mathbf{M} \times \mathbf{B} dv_{\mathrm{mt}}, \qquad \text{(s1.1)}$$

where $\vec{\tau}_{\mathrm{d}}$ is the deflection torques for the magnetic tail, v_{mt} is the volume of the magnetic tail, **B** is the flux density of the applied field and **M** is the magnetization of the magnetic tail (the initial magnetic domain state is horizontal and along the tail's axial direction). Combing with an applied magnetic field generated by the Helmholtz coil, the magnetic torques developed on the magnetic tail for deflection are expressed as follows,

$$|\vec{\tau}_{\mathrm{d}}| = \int_{v_{\mathrm{mt}}} |\mathbf{M}|\,|\mathbf{B}| \sin\left(\frac{\pi}{2} - \varphi\right) \cos\big(f(dv_{\mathrm{mt}})\big)\, dv_{\mathrm{mt}}, \qquad \text{(s1.2)}$$

where φ is the angle between **B** and $\mathbf{B}_z$, $f(dv_{\mathrm{mt}})$ represents the angle between the **M** at dv_{mt} and horizontal plane, and this angle is determined by the positions and postures of dv_{mt}.

During the whole manipulation process, the Uni-SoPro will turn parallel to $\mathbf{B}_{x\text{-}y}$ very quickly according to the abovementioned orientation oscillation results (generally less than 25 ms at 10 mT). It means the vector **B** and vector **M** will always be in the same plane. Then, Eq. s1.2 can be expressed as,

$$|\vec{\tau}_{\mathrm{d}}| = \int_{v_{\mathrm{mt}}} |\mathbf{M}|\,|\mathbf{B}_{\mathrm{z}}| \cos\big(f(dv_{\mathrm{mt}})\big)\, dv_{\mathrm{mt}}. \tag{s1.3}$$

Since the **M** profiles of the magnetic tail are fixed after magnetization, the magnetic torques $|\vec{\tau}_{\mathrm{d}}|$ for magnetic tail vertical deflection is only dependent on the amplitude of $\mathbf{B}_z$. Thus, Eq. s1.3 can be further simplified as,

$$|\vec{\tau}_{\mathrm{d}}| \sim |\mathbf{B}_{\mathrm{z}}|. \tag{s1.4}$$

Hence, the fuel release and propulsion control of Uni-SoPros, are inherently independent on $\mathbf{B}_{x\text{-}y}$, and determined solely by the magnetic field component $\mathbf{B}_z$.

For direction control of Uni-SoPros, steering chips (SCs) that are made of magnetic m-PDMS are embedded in the main body of the Uni-SoPro. The magnetization profiles of SCs are the same as the magnetic tail. In fact, during the magnetically induced orientation process, the magnetic tail also plays a significant synergistic role (Noticing that the whole Uni-SoPro is unstrained in horizontal rotation, the base of the magnetic tail is fixed in the deflection characterization. The rotation resistance can be considered far less than the deflection residence in this case. this part of horizontal magnetic torque actually will act on the steering process of the whole Uni-SoPro rather than leading to a horizontal deflection of the tail). The magnetic torques for the orientation can be expressed as,

$$\vec{\tau}_{\mathrm{o}} = n \int_{v_{\mathrm{sc}}} \mathbf{M} \times \mathbf{B}\, dv_{\mathrm{sc}} + \int_{v_{\mathrm{mt}}} \mathbf{M} \times \mathbf{B}\, dv_{\mathrm{mt}}, \tag{s1.5}$$

where $\vec{\tau}_{\mathrm{o}}$ is the orientation torques for the soft propulsor, v_{sc} is the volume of the magnetic SC, *n* represents the number of the steering chips (in this work, n=2 is selected for the most cases). Assuming that the Uni-SoPro is initially placed on the water surface along the *x-axis*, combing the

applied magnetic field generated by the Helmholtz coil, the magnetic torques developed on the Uni-SoPro for orientation are expressed as,

$$|\vec{\tau}_\mathrm{o}| = n\int_{v_\mathrm{sc}} |\mathbf{M}|\,|\mathbf{B}|\sin\varphi\sin\theta\,dv_\mathrm{sc} + \int_{v_\mathrm{mt}} |\mathbf{M}||\mathbf{B}|\sin\varphi\sin\theta\cos\big(f(dv_\mathrm{mt})\big)\,dv_\mathrm{mt}, \quad \text{(s1.6)}$$

where φ is the angle between **B** and $\mathbf{B}_\mathrm{z}$, θ is the angle between the horizontal projection ($\mathbf{B}_\text{x-y}$) of **B** and x-axis, and $f(dv_\mathrm{mt})$ is the angle between the **M** at dv_mt and the horizontal plane. According to the orthogonal relationship of the 3D magnetic field, Eq. s1.6 can be expressed as,

$$|\vec{\tau}_\mathrm{o}| = n\int_{v_\mathrm{sc}} |\mathbf{M}|\,|\mathbf{B}_\mathrm{x-y}|\sin\theta\,dv_\mathrm{sc} + \int_{v_\mathrm{mt}} |\mathbf{M}||\mathbf{B}_\mathrm{x-y}|\sin\theta\cos\big(f(dv_\mathrm{mt})\big)\,dv_\mathrm{mt}. \quad \text{(s1.7)}$$

The **M** profiles of SCs and magnetic tail are frozen after magnetization, for a given $\mathbf{B}_\text{x-y}$, the θ is also determined. Therefore, the magnetic torques $|\vec{\tau}_\mathrm{o}|$ for the orientation of the Uni-SoPro is determined. Thus, Eq. s1.7 can be further simplified as,

$$|\vec{\tau}_\mathrm{o}| \sim |\mathbf{B}_\mathrm{x-y}|. \quad \text{(s1.8)}$$

Hence, the orientation control of the Uni-SoPro is inherently independent on $\mathbf{B}_\mathrm{z}$, and determined solely by the magnetic field component $\mathbf{B}_\text{x-y}$. In summary, the propulsion and direction control of Uni-SoPros are dependent on $\mathbf{B}_\mathrm{z}$ and $\mathbf{B}_\text{x-y}$, respectively. In a practical operation, a resultant 3D magnetic field **B** can achieve agile motion manipulation of the Uni-SoPro.

Supplementary Note 2. Dynamics analysis of basic propulsion states

The Uni-SoPro's kinetic characteristics is fundamentally determined by all the forces acting on it. It is necessary to analyze and calculate these forces to further comprehend the fundamental sciences behind the swift and agile characteristics of the Uni-SoPro. Referring to the previous literature (*2*), we believe the following forces may major affect its kinetic characteristics: hydrostatic force, viscosity, tension, wave resistance, and momentum. The resultant force F can be described in Eq. s2.1,

$$F = \int \underbrace{\rho_\mathrm{f} g h(x) l(x)\sin\beta\,dx}_{\text{hydrostatic}} + \underbrace{\mu_\mathrm{f} S\frac{dv}{dz}\cos\beta}_{\text{viscosity}} - \underbrace{\int_C \gamma\mathbf{t}\frac{\mathbf{v}}{|\mathbf{v}|}dl}_{\text{tension}} + \underbrace{R_\mathrm{W}}_{\text{wave resistance}} - \underbrace{\rho_\mathrm{f} S_1 (v_c - v)^2\sin\beta}_{\text{momentum}}. \quad (s2.1)$$

In this section, we will first collate, deduce and simplify the formulation of each part of the force in different states. Then, we focus on the braking process and calculate the forces in typical states. In this section, we mainly focus on the horizontal component of forces.

2.1 *Hydrostatic force*

The Uni-SoPro's bottom contacts with water all the time, and water pressure keeps acting on it. When the Uni-SoPro tilts on water, such an asymmetry will lead to a force horizontally. It can be described as,

$$F_{\mathrm{H}} = \int \rho_{\mathrm{f}} g h(x) l(x) \sin\beta \, dx, \qquad \text{(s2.2)}$$

where ρ_{f} is the density of water, g is the gravity acceleration, $h(x)$ is the depth at x, $l(x)$ is the length at x, and β is the angle between the bottom and horizontal plane. To simplify the calculation, we ignore the deformation of the bottom. We can express $h(x)$ by,

$$h(x) = h_{\max} - x \sin\beta, \qquad \text{(s2.3)}$$

where $h_{\max}$ is the $h(x)$ of the deepest point (Supplementary Fig 21a).

2.2 *Viscous force*

Viscous force is a resident force when it comes to relative motion between solid items and liquid. The Uni-SoPro is also acted by viscous force,

$$F_{\mathrm{V}} = \mu_{\mathrm{f}} S_{\mathrm{wet}} \frac{dv}{dz_n} \cos\beta, \qquad \text{(s2.4)}$$

where μ_{f} is the viscosity of water, S_{wet} is the wetting area of the bottom, v is the relative velocity of Uni-SoPro to the water, z_{n} is the normal distance to the bottom, and dv/dz_{n} is the velocity gradient at the bottom surface of the Uni-SoPro. We usually substitute it with

$$\frac{dv}{dz_n} = \frac{v \cos\beta}{\delta}, \qquad \text{(s2.5)}$$

where δ is the boundary layer thickness, which is calculated from the equation:

$$\delta = \frac{l}{\sqrt{\mathrm{Re}}}, \qquad \text{(s2.6)}$$

where Re is Reynolds number, l is the length of the Uni-SoPro. Among them, Re is expressed as,

$$\mathrm{Re} = \frac{\rho_{\mathrm{f}} v l \cos\beta}{\mu_{\mathrm{f}}}. \tag{s2.7}$$

Associating Eq. s2.4 to Eq. s2.7, then we can calculate the viscous force F_V.

2.3 *Surface tension gradient*

Surface tension acts on the interphase between the Uni-SoPro and water, which is the premise of Marangoni effect. It is vertical to the outline of the Uni-SoPro and tangent to water surface. The sum of the surface tension can be written as,

$$F_{\mathrm{T}} = \int_C \gamma \mathbf{t} \frac{\mathbf{v}}{|\mathbf{v}|} dl, \tag{s2.8}$$

where γ is the magnitude of the tension locally, $\mathbf{t}$ is the unit vector of the direction of tension. $\mathbf{v}$ is the velocity of the Uni-SoPro. Actually, the motion-induced distortion of water surface and the release of NOP would both influence this force. To simplify it in a scalar form, the tension tensor can be described by the three parameters: $\theta_t(x)$, $\varphi_t(x)$, and $\gamma(x)$, where x is the coordinate along the edge beginning from point A, $\theta_t(x)$ denotes the included angle between the horizontal projection of the normal line of point x at the contact line and the positive x-axis, $\varphi_t(x)$ describes the included angle between normal line of point x at the contact line and horizontal plane, and $\gamma(x)$ is the magnitude of the tension at point x which can be tuned by the release of NOP (Supplementary Fig 21b). The horizontal force is,

$$F_{\mathrm{T}} = \int_C \gamma(x) \cos\varphi_{\mathrm{t}}(x) \cos\theta_{\mathrm{t}}(x)\, dx\,. \tag{s2.9}$$

Such an integral makes it hard for measuring $\varphi_{\mathrm{t}}(x)$ in a distorted water surface. To simplify the calculation, a few measures are adopted here: a) divide the outline of the Uni-SoPro with two wings' points; b) assume φ_t will not change along the outline; c) measure the φ_t at two ends of the Uni-SoPro (φ_{head} and φ_{tail}); and d) replace $\varphi_t(x)$ with two measurable parameters φ_{head} and φ_{tail}. Based on the above simplifications, it can be considered a curvilinear integral. Thus, we can calculate the horizontal force with,

$$F_{\mathrm{T}} = l_w \gamma_0 (\cos\varphi_{\mathrm{head}} - \cos\varphi_{\mathrm{tail}}), \tag{s2.10}$$

where l_w is the span of the wings of Uni-SoPro, γ_0 is the magnitude of the tension of water at 20°C. However, such a simplification will also lead to some errors. Here, we employed two correction

factors k_1 and k_2 to revise Eq. s2.10. The distribution of $\varphi_t(x)$ along the outline of the Uni-SoPro can be made up via proper k_1 and k_2. The equation is,

$$F_T = l_w\gamma_0(\cos(k_1\varphi_{head}+k_2\varphi_{tail}) - \cos(k_1\varphi_{tail} + k_2\varphi_{head})). \quad (s2.11)$$

As the above calculation ignores the influence of NOP, it will change the magnitude of the tension water from γ_0 to γ_1. The equation for the cruise state is,

$$F_T = l_w\gamma_0(\cos(k_1\varphi_{head}+k_2\varphi_{tail}) - \cos(k_1\varphi_{tail} + k_2\varphi_{head})) + l_t(\gamma_0 - \gamma_1)\cos(k_1\varphi_{tail} + k_2\varphi_{head}), \quad (s2.12)$$

where l_t is the span of the caudal fin. In this section, k_1, k_2 are set as 5/9 and 4/9.

2.4 *Momentum induced by convection*

When the magnetic tail dips into the surface, NOP is released onto the water, and a drastic convection is induced. Water is moving fast away from the convective center, and some of the water that flow to Uni-SoPro will be blocked by the caudal fin of the Uni-SoPro (Fig. 4a). This phenomenon results in forward momentum transfer to the Uni-SoPro. The momentum can be calculated as follows,

$$F_M = \rho_f S_1(v_c - v)^2 \sin\beta, \quad (s2.13)$$

where ρ_f is the density of the fluid, S_1 is the projected area of momentum transfer, v is the velocity of Uni-SoPro. Momentum only occurs when the tail is in the water, we measured the convection velocity v_c by conspicuous floating particles.

2.5 *Wave resistance*

Wave resistance is a common concept in ship design, which is resistance that comes from wave generation. When Uni-SoPro moves on water, the motion also disturbs the free surface and forms waves on the water. So, we believe there is a similar resistance acting on Uni-SoPro. According to the wave characteristics (*3*), the wave resistance is considered as,

$$R_{wp} = \frac{1}{4}\rho_f g A^2\left[1 - \frac{4\pi h}{L_{wave}}(\sinh\frac{4\pi h}{L_{wave}})^{-1}\right], \quad (s2.14)$$

where R_{wp} is the wave resistance per unit length of the wave, A is the amplitude of the wave, h is the depth of the water, and L_{wave} is the wavelength. Through measurement, h is 15 mm, and thus L_{wave} is 15 mm. Therefore, $sinh 4\pi$ is a large number. The terms in brackets can be considered as 1 (*sinh* here indicates the hyperbolic sine function). Thus, it can be further simplified as,

$$R_{\mathrm{wp}} = \frac{1}{4}\rho_{\mathrm{f}} g A^2. \tag{s2.15}$$

Considering A to be the same along wave width, we take wave width (w_{wave}) as l, and l is the width of the Uni-SoPro. Therefore, the wave resistance is,

$$R_{\mathrm{w}} = \frac{1}{4}\rho_{\mathrm{f}} g A^2 l. \tag{s2.16}$$

Based on the above forces, we can analyze the movement of the Uni-SoPro. First, Newton's second law is introduced,

$$F = m\frac{d^2 x_c}{dt^2}. \tag{s2.17}$$

It builds up a bridge between force and dynamic performance.

2.6 *Force analysis in typical states*

The agility of the Uni-SoPro relies largely on braking behavior. We divide the whole braking process into four typical states: cruising state (S1), triggering state (S2), braking state (S3), and titling forward state (S4). In this section, we calculated each force in these four typical states.

2.6.1 *Hydrostatic force*

To calculate the hydrostatic force, we need to measure l(x) which describes the outline of the Uni-SoPro. l(x) is extracted from the designed contour of the bottom in MATLAB. We measured $h_{\max}$ and β at the frames in the video in side view according to the four states defined. The hydrostatic force in four states (F_{H1}, F_{H2}, F_{H3}, and F_{H4}) are calculated as:

$$\begin{cases} F_{\mathrm{H1}} = 2.116 \times 10^{-6}\, N, & (h_{max}, \beta) = (1.5, 22.4) \\ F_{\mathrm{H2}} = 2.116 \times 10^{-6}\, N, & (h_{max}, \beta) = (1.5, 22.4) \\ F_{\mathrm{H3}} = 0\, N, & (h_{max}, \beta) = (0.0, 0.0) \\ F_{\mathrm{H4}} = -5.954 \times 10^{-6}\, N, & (h_{max}, \beta) = (2.2, -21) \end{cases}. \tag{s2.18}$$

2.6.2 *Viscosity*

The parameters for calculating viscosity are measured: v=200 mm/s, l=7.2 mm, β=20°, μ_{f}=1.01×10^{-3} Pa·s. According to Eq. s2.7, Re=1340<2000, which means the flow below can be considered as a laminar flow. Therefore, we can obtain the thickness of the boundary layer:

δ=1.97×10^{-4} m. The velocity gradient can be approximately calculated with Eq. s2.5. Considering that the whole bottom is wet, S_{wet} is equal to S, which is measured to be around 19.77 mm^2. We can calculate the viscosity of the four states of braking,

$$\begin{cases} F_{\mathrm{V1}} = 1.836 \times 10^{-5}\, N \\ F_{\mathrm{V2}} = 1.836 \times 10^{-5}\, N \\ F_{\mathrm{V3}} = 2.582 \times 10^{-6} N \\ F_{\mathrm{V4}} = 1.104 \times 10^{-6}\, N \end{cases}. \quad \text{(s2.19)}$$

2.6.3 *Surface tension gradient*

When the temperature is around 20°C, γ_0 is 72 mN/m. We measure the actual γ where the water is dipped by NOP to be γ_1, and γ_1 is 57 mN/m. The span of the wings l_w is 7.2 mm, and the span of the caudal fin l_t is 2.5 mm. The surface tension forces are,

$$\begin{cases} F_{\mathrm{T1}} = 3.279 \times 10^{-5}\, N, \quad (\varphi_{\mathrm{head}}, \varphi_{\mathrm{tail}}) = (25, 20) \\ F_{\mathrm{T2}} = -1.924 \times 10^{-6}\, N, \ \ (\varphi_{\mathrm{head}}, \varphi_{\mathrm{tail}}) = (25, 20) \\ F_{\mathrm{T3}} = -3.100 \times 10^{-5}\, N, \ \ (\varphi_{\mathrm{head}}, \varphi_{\mathrm{tail}}) = (64, 16) \\ F_{\mathrm{T4}} = -5.283 \times 10^{-5}\, N, \ \ (\varphi_{\mathrm{head}}, \varphi_{\mathrm{tail}}) = (81, 0) \end{cases}. \quad \text{(s2.20)}$$

2.6.4 *Momentum induced by convection*

Here, v_c is measured as around 350 mm/s, v is around 200 mm/s. S_1 is the area where momentum acts, and it's estimated based on the underwater area of the caudal fins. It is estimated to be 1.95 mm^2. Then, we obtain,

$$F_{\mathrm{M}} = 5.1 \times 10^{-6}\, N. \quad \text{(s2.21)}$$

2.6.5 *Wave resistance*

We measure the amplitude A of the four states,

$$\begin{cases} A_1 = 6.40 \times 10^{-4}\, m \\ A_2 = 8.20 \times 10^{-4}\, m \\ A_3 = 1.25 \times 10^{-3}\, m \\ A_4 = 7.20 \times 10^{-4}\, m \end{cases}, \quad \text{(s2.22)}$$

and the wave resistance can be calculated,

$$\begin{cases} R_{\mathrm{w}1} = 3.63 \times 10^{-6}\ N \\ R_{\mathrm{w}2} = 5.90 \times 10^{-5}\ N \\ R_{\mathrm{w}3} = 2.76 \times 10^{-5}\ N \\ R_{\mathrm{w}4} = 9.15 \times 10^{-6}\ N \end{cases}. \tag{s2.23}$$

The results are plotted into a histogram (Fig. 4e). We can see from above that the rapid braking of the Uni-SoPro mainly comes from the wave resistance and the tension difference of the water surface, but other terms are also non-negligible. The tension differences in braking states indicate a difference in contact angle between the front and the rear of Uni-SoPro.

2.7 *Braking analysis with different magnetic fields*

According to our analysis in Fig. 4e, wave resistance and tension difference induced by distortion of water surface play dominative roles in the braking process. These two terms are largely related to the flipping action and tilting-forward action of the Uni-SoPro. For the wave resistance, the amplitude of the wave can be larger when the flipping action is stronger and faster. For the tension differences, larger water surface distortion may cause a greater horizontal tension difference. To verify that, we measured and calculated the wave resistance and the tension differences in the braking process, under the 100 Gs braking magnetic field with different directions, which will influence the trajectory and posture changing during the braking process. We applied braking magnetic fields with included angles of φ=10°, 45° and 60°, respectively (Fig. 4b). In addition, to keep the comparison fair, all of the initial horizontal speeds were kept at about 200 mm/s. According to the experimental and estimated results, these terms could be calculated as follows.

For tension gradient,

$$\begin{cases} F_{\mathrm{T}10} = -6.984 \times 10^{-5}\ N \quad (\varphi_{\mathrm{head}}, \varphi_{\mathrm{tail}}) = (99, 17) \\ F_{\mathrm{T}45} = -4.944 \times 10^{-5}\ N \quad (\varphi_{\mathrm{head}}, \varphi_{\mathrm{tail}}) = (80, 11) \\ F_{\mathrm{T}60} = -3.909 \times 10^{-5}\ N \quad (\varphi_{\mathrm{head}}, \varphi_{\mathrm{tail}}) = (69, 2) \end{cases}. \tag{s2.24}$$

For wave resistance,

$$\begin{cases} A_{10} = 1.55 \times 10^{-4}\ m \\ A_{45} = 1.01 \times 10^{-4}\ m \\ A_{60} = 0.82 \times 10^{-3}\ m \end{cases}, \tag{s2.25}$$

$$\begin{cases} R_{\mathrm{w}10} = 8.476 \times 10^{-5}\ N \\ R_{\mathrm{w}45} = 3.599 \times 10^{-5}\ N \\ R_{\mathrm{w}60} = 2.372 \times 10^{-5}\ N \end{cases}. \tag{s2.26}$$

The results agree well with our assumptions above. To reach better instantaneous braking performance, it looks like we should keep the angle φ as small as possible. However, too small angle φ will also bring a larger velocity bounce according to the experimental results in Fig. 4d. To trade off these influences, we usually use the braking magnetic field of 45° to manipulate the Uni-SoPros.

Supplementary Note 3. **Scaling analysis of the kinetic performance**

Fig. 3d shows that the peak velocity of the Uni-SoPro is related to its characteristic length scale (C_{bl}). We further analyze the scaling law of the Uni-SoPro based on Eq. s2.1. Only horizontal components are considered in the following,

$$0 = \int \rho_{\mathrm{f}} g h(x) l(x) \sin\beta \, dx + \mu_{\mathrm{f}} S \frac{dv}{dy} |\sin\beta| - \int_C \gamma \mathbf{t} \frac{\mathbf{v}}{|\mathbf{v}|} dl + F_{\mathrm{W}} - \rho_{\mathrm{f}} S_1 (v_c - v)^2 \sin\beta \,. \quad \text{(s3.1)}$$

For hydrodynamic pressure, F_{H} has the form of,

$$F_{\mathrm{H}} = \int \rho_{\mathrm{f}} g h(x) l(x) \sin\beta \, dx \,. \quad \text{(s3.2)}$$

Supposing that F_{H} is positively correlated with the third power of C_{bl}, according to the results in Supplementary Note 2, the hydrodynamic pressure can be approximated as,

$$F_{\mathrm{H}} = k_{\mathrm{h}} {C_{\mathrm{bl}}}^3, \quad \text{(s3.3)}$$

where k_{h} is the scale factor and k_{h}=5.6.

For viscosity force, F_{V}, supposing that the liquid here is incompressible, F_{V} can be expressed as follows,

$$F_{\mathrm{V}} = \mu_{\mathrm{f}} S_{\mathrm{wet}} \frac{dv}{dy} \cos\beta \,, \quad \text{(s3.4)}$$

where μ_{f} is the dynamic viscosity of the fluid, v is the velocity component in the horizontal direction, y is the coordinate of the flow field in the vertical directions, α is the angle between the bottom and horizontal plane, and $S_{\mathrm{wet}}=0.37C_{\mathrm{bl}}^2$ is the wetting area of the propulsor. According to the boundary layer theory, the change of velocity in the vertical direction can be ignored compared with the horizontal direction. Assuming that v is linearly distributed in the boundary layer and ignoring the bottom liquid velocity,

$$F_{\mathrm{v}} = \mu_{\mathrm{f}} S_{\mathrm{wet}} \frac{v \cdot \cos\beta}{\delta} \cos\beta\,, \qquad \text{(s3.5)}$$

where δ is the thickness of the boundary layer(*4*),

$$\delta \sim \frac{C_{\mathrm{bl}}}{\sqrt{\mathrm{Re}}}, \qquad \text{(s3.6)}$$

where Re is the Reynolds number. Therefore, hydrodynamic force F_{v} can be simplified as follows,

$$F_{\mathrm{v}} = \mu_{\mathrm{f}} S_{\mathrm{wet}} \frac{v}{\delta} = -0.37 \cdot (\cos\beta)^2 \sqrt{\rho_{\mathrm{f}} \mu_{\mathrm{f}} \cos\beta} \cdot v^{\frac{3}{2}} \cdot C_{\mathrm{bl}}{}^{\frac{3}{2}}. \qquad \text{(s3.7)}$$

For tension difference, F_{T} can be expressed as follows,

$$F_{\mathrm{T}} = \int_C \gamma \boldsymbol{t} \frac{\boldsymbol{v}}{|\boldsymbol{v}|} dl = \int_C \gamma(x) \cos\varphi_{\mathrm{t}}(x) \sin\theta_{\mathrm{t}}(x)\, dx\,. \qquad \text{(s3.8)}$$

When in the cruising state, the release of NOP induced a decrease of γ from γ_0 to γ_1. The boundary between two tail points is where γ_1 acts and the span of it is l_{t}. Hence, the equation will be,

$$F_{\mathrm{T}} = l_w \gamma_0 (\cos(k_1 \varphi_{\mathrm{head}} + k_2 \varphi_{\mathrm{tail}}) - \cos(k_1 \varphi_{\mathrm{tail}} + k_2 \varphi_{\mathrm{head}})) + l_{\mathrm{t}} (\gamma_0 - \gamma_1) \cos(k_1 \varphi_{\mathrm{tail}} + k_2 \varphi_{\mathrm{head}})\,, \qquad \text{(s3.9)}$$

where l_{w} is the span of the wings as the length where the tension acts.

For wave resistance F_{W}, the wavelength (L_{wave}) of the wave excited by the movement of the Uni-SoPro is about 4 mm. According to the wave theory, $h_{\mathrm{water}} > L_{\mathrm{wave}}/2$, where h_{water} is the depth of experimental water (~20 mm), so the excited wave is considered as a deep-water wave(*5*), and its velocity can be written as,

$$C_{\mathrm{wave}} \sim \sqrt{\frac{g L_{\mathrm{wave}}}{2\pi}}, \qquad \text{(s3.10)}$$

where C_{wave} is the velocity of the excited wave, substituting g=9.8 m/s^2, L_{wave}=4 mm into Eq. s3.9 can obtain C_{wave}~250 mm/s. Here, only the resistance corresponding to the energy consumed by generating waves is considered. According to (*3*), F_{t} can be written as,

$$F_{\mathrm{W}} \sim \frac{1}{4} \rho_{\mathrm{f}} g A^2 \cdot w_{\mathrm{wave}}, \qquad \text{(s3.11)}$$

where A is the amplitude of the wave, w_{wave} is the width of the wave. Considering A to be the same along wave width, A can be approximated as ~0.09C_{bl} when the characteristic length scale is small (C_{bl}<2 cm) and w_{wave} is ~C_{bl}.

For momentum, F_M is calculated as follows,

$$F_M = \rho_f S_1 (v_c - v)^2 \sin\beta \,, \tag{s3.12}$$

where S_1 is the area of where momentum acts, v_c is the convection velocity. During the cruising state, v_c is ~350 mm/s, and S_1 is ~$3.76\times10^{-5}C_{bl}^2$. According to the above approximation and simplification, Eq. s3.1 in such a particular case can be written as follows,

$$\begin{gathered} k_h C_{bl}{}^3 - 0.37 \cdot (\cos\beta)^2 \sqrt{\rho_f \mu_f \cos\beta} \cdot v^{\frac{3}{2}} \cdot C_{bl}{}^{\frac{3}{2}} + \\ l_w \gamma_0 (\cos(k_1 \varphi_{head} + k_2 \varphi_{tail}) - \cos(k_1 \varphi_{tail} + k_2 \varphi_{head})) \\ + l_t (\gamma_0 - \gamma_1) \cos(k_1 \varphi_{tail} + k_2 \varphi_{head}) + \frac{1}{4} \rho_f g A^2 \cdot w_{wave} + \rho_f S_1 (v_c - v)^2 \sin\beta = 0, \end{gathered} \tag{s3.13}$$

where k_h=5.6, β=20°, φ_{head}=25°, φ_{tail}=20°, γ_1=5.7×10^{-2} N/m, γ_0=7.2×10^{-2} N/m, l_w=C_{bl}, l_t=$0.35C_{bl}$, k_1=5/9, k_2=4/9 ρ_f=10^3 kg/m^3, μ_f=1.01×10^{-3} Pa·s (20°C), A=$0.09C_{bl}$ and w_{wave}=C_{bl}. Substituting the above values, Eq. 3.12 can be written as,

$$6 \cdot C_{bl}{}^3 - 0.318 \cdot v^{\frac{3}{2}} \cdot C_{bl}{}^{\frac{3}{2}} + 5.565 \times 10^{-3} C_{bl} + 19.845 C_{bl}{}^3 + 0.0129(0.35 - v)^2 C_{bl}{}^2 = 0. \tag{s3.14}$$

As in Fig. 3d and Supplementary Table 5, the calculated result shows the same trend as the measured speed. It can be seen that our calculated value is slightly smaller than the actual value, which may be due to the overestimation of the viscosity force and the traveling wave resistance at a small scale.

In addition, we carried out duration experiments on the Uni-SoPros. We employed a Uni-SoPro with a scale of 1.2, enabling it to execute reciprocal motion within a water tank measuring approximately 18 cm × 28 cm × 3.5 cm. Remarkably, it exhibited a runtime of 19.4 minutes, covering a cumulative distance exceeding 110 meters. It's worth noting that this duration experiment took place in a confined water environment without water renewal. In a more open water setting, its endurance capacity is theoretically even greater.

Supplementary Note 4. Surfactant release study by finite element analysis

The local release of surfactant plays a significant role in Marangoni propulsion. Hence, the delivery strategy (e.g., fuel delivery function) will directly influence the propulsion behaviors and manipulation windows. Here, a finite element analysis was used to compare the surfactant spread process with different delivery strategies. According to the results of Fig. 2f, the tail with inserted

fibres shows an obviously slower attenuation of local surface tension tuning capability than that without fibres, especially short trigger time was adopted. Here, we used a linear attenuation function to describe the surfactant concentration of the touching point of the tail. The concentration function can be expressed as,

$$c(t) = c_{\mathrm{t}} - \varepsilon t, \tag{s4.1}$$

where c_{t} is the initial concentration of touching point, ε is the attenuation coefficient (which is determined by different magnetic tail and trigger mode). The surface tension of water is a function of the concentration of NOP(*6*), and it can be expressed as,

$$\gamma(c) = -20\,\mathrm{lg}\,c + 52, \tag{s4.2}$$

where $\gamma(c)$ represents the surface tension, c is surfactant concentration. When the $c<1$ mol/m^3, Eq. s4.2 can be fitted by a linear function as follows,

$$\gamma(c) = \gamma_{\mathrm{w}} - \eta c, \tag{s4.3}$$

where γ_w is the surface tension of DI water at 20°C (~72 mN/m), η is tension coefficient associated with c. The η can be determined as 0.03 N·m^2/mol by a linear fitting. Therefore, Eq. s4.3 can be expressed as follows,

$$\gamma(c) = 0.072 - 0.02c. \tag{s4.4}$$

There would be a surface tension gradient caused by a concentration gradient if the tail touched the water surface locally, which would induce a Marangoni flow in the water surface. Meanwhile, due to the concentration gradient, there will be a diffusion behavior as well. The molecule behavior can be described by,

$$\frac{\partial c_{\mathrm{i}}}{\partial t} + \nabla \cdot J_{\mathrm{i}} + \mathrm{u} \cdot \nabla c_{\mathrm{i}} = R_{\mathrm{i}}, \tag{s4.5}$$

$$J_{\mathrm{i}} = -D_{\mathrm{i}} \nabla c_{\mathrm{i}}, \tag{s4.6}$$

where the diffusion coefficient of NOP in water D_{i} was estimated as 1×10^{-9} m^2/s approximately by checking typical diffusion coefficients (usually between 10^{-9}-10^{-10} m^2/s for small molecules)[a]. A contour of the Uni-SoPro with a sized scale of 2.4 was imported for considering fluid-solid coupling behavior in a limited water area (width: 0.2 m, length: 0.3 m, height: 0.02 m). The

[a] In fact, the effect of diffusion behavior is very weak, and hence this coefficient has little effect on the concentration distribution in a very short time.

touching point that the magnetic tail delivers surfactant onto the water surface was considered an inflow, which is a function of time described as Eq. s4.1.

Here, three concentration function of touching point were selected for comparing migration process of surfactant (c_t=0.5 mol/m^3, ε=0.033 mol/(m^3·s); c_t=0.6 mol/m^3, ε=0.13 mol/(m^3·s); c_t=0.7 mol/m^3, ε=0.2 mol/(m^3·s)).

By extracting the surfactant concentration of the head point and tail point at the contacting line, respectively, we can observe that a large initial concentration gradient between the head point (0, 200 mm) and tail point (0, 181 mm) at the contacting line can be obtained, with a high initial concentration c_t with high attenuation ε. However, as the gradual molecule accumulation in the limited water and the attenuation of concentration at the touching point, the concentration gradient would dramatically decrease, which is not contributed to stable propulsion. By contrast, the inflow at the touching point with a low attenuation coefficient can provide a more stable concentration gradient and surface tension gradient between the head point and tail point. Therefore, the surfactant delivery strategy with a low attenuation coefficient is preferred, since it can provide a relatively stable propulsion force, and hence enable a broader operation widow for the manipulation of Uni-SoPros.

Supplementary Note 5. Halt simulations

To further understand and illustrate the braking mechanisms of the Uni-SoPros, we built a model to analyze its hydrodynamics. Utilizing a computational fluid dynamics (CFD) method, we simulated the whole moving process to analyze its hydrodynamic drag and energy transfer.

Specifically, a six-DOF model was employed to compute and analyze the kinetic characterizations of Uni-SoPros. In such a model, the magnetically/hydrodynamically induced deformation of the Uni-SoPro was ignored and the whole body was considered rigid, but the elastic effect was still considered in the load condition. The mass, moment of inertia, magnetic moment, Marangoni force in each motion state were set also in the user-defined functions (UDF). These boundary and load conditions were set according to the experimental conditions.

Remeshing of fluid meshes, smoothing and remeshing dynamic mesh technologies were applied for preventing negative mesh, where spring constant factor was set as 0.5, convergence

tolerance was set as 0.0001, minimum length scale was set as 0.04 and maximum length scale was set as 0.4.

Definition of fluid/air, a two-phase (water and air, respectively) model (VOF, volume of fluid) was employed, where the surface tension coefficient was defined as 0.07 N/m. In addition, an RNG k-ε (2 eqn) model with standard wall function near-wall treatment was employed, where model constants C_{mu}=0.085, C1-ε=0.085, C2-ε=1.68.

Definition of the basic physical properties of Uni-SoPro, the mass, moment of inertia and center of gravity location (*CGL*) of Uni-SoPro were measured and given as,

$$\begin{cases} Mass = 1.86 \times 10^{-5}\ kg \\ I_{xx} = 2.729\ \times 10^{-11}\ kg \cdot \mathrm{m}^2 \\ I_{yy} = 5.706\ \times 10^{-11}\ kg \cdot \mathrm{m}^2 \\ I_{zz} = 6.209 \times 10^{-11}\ kg \cdot \mathrm{m}^2 \\ CGL = (1.503258, 0, 1.6524893) \end{cases} . \tag{s5.1}$$

Besides these conditions, the translation motion on the *y*-axis and rotation motion on the *x*- and *z*-axis were also constrained.

Magnetic moment for tilt controlling, the magnetic moment consists of two parts, magnetic tail-induced torque, and steering chips-induced torque, respectively. We estimated the whole torque as follows,

$$\vec{\tau}_{\mathrm{w}} = \int_{v_{\mathrm{mt}}} \mathbf{M} \times \mathbf{B} dv_{\mathrm{mt}} + 2 \int_{v_{\mathrm{mt}}} \mathbf{M} \times \mathbf{B} dv_{\mathrm{sc}}, \tag{s5.2}$$

where $\vec{\tau}_{\mathrm{w}}$ is the whole torques for tilt controlling of Uni-SoPro. Since the tilting angle of Uni-SoPro is changing at different stages, the magnetic torque is highly dependent on this tilting angle and applied magnetic field, and it can be written as,

$$\begin{cases} \vec{\tau}_{\mathrm{w}} = |\mathbf{M}||\mathbf{B}|(v_{\mathrm{mt}} + 2v_{\mathrm{sc}}) \sin\left(-\frac{\pi}{3} - \sigma\theta\right), \text{Accelartion states} \\ \vec{\tau}_{\mathrm{w}} = |\mathbf{M}||\mathbf{B}|(v_{\mathrm{mt}} + 2v_{\mathrm{sc}}) \sin\left(\frac{\pi}{4} - \sigma\theta\right), \text{Braking states} \end{cases} , \tag{s5.3}$$

where σ correction factor for compensating the deformation of tail deflection and small deformation of steering chips, in this analysis, σ takes 2.1. According to our magnetization characterization results on the used magnetic materials, **M** takes 125 kA/m, and the applied external magnetic field **B** is 100 Gs. And the volume of magnetic parts can be estimated as,

$$\begin{cases} v_{\mathrm{mt}} = \pi(t_c^2 - (t_c - d_t)^2)L \\ v_{\mathrm{sc}} = D^2 t_{body} \end{cases}, \quad (s5.4)$$

where t_c is the caliber of the magnetic tail, d_t is the thickness of the magnetic tail, *L* is the length of the magnetic tail, *D* is the side length of steering chips, and t_{body} is the thickness of the steering chips. According to our design parameters, v_{mt} is estimated as 1.44× 10^{-10} m^3 and $2v_{\mathrm{sc}}$ is estimated as 1.28×10^{-10} m^3. In addition, the magnetic tail is an elastic energy storage/release element (a compliant mechanism) during the dynamic process. Here, we consider it as a torsion spring in the whole system. Therefore, an elastic term was introduced to consider this behavior. The whole process is divided into four states, and each state can be written as,

$$\begin{cases} \vec{\tau}_{\mathrm{w}} = 3.4\sin\left(-\frac{\pi}{3} - 2.1\theta\right) - 30\theta, (10^{-7}\mathrm{N\cdot m}), \text{Trigger phase } (0, 0.01\mathrm{s}) \\ \vec{\tau}_{\mathrm{w}} = 3.4\sin\left(-\frac{\pi}{3} - 2.1\theta\right), (10^{-7}\mathrm{N\cdot m}), \text{Accelartion phase } (0.01, 0.035\mathrm{s}) \\ \vec{\tau}_{\mathrm{w}} = 3.4\sin\left(\frac{\pi}{4} - 2.1\theta\right) - 30\theta, (10^{-7}\mathrm{N\cdot m}), \text{Braking phase } (0.035, 0.05\mathrm{s}) \\ \vec{\tau}_{\mathrm{w}} = 3.4\sin\left(\frac{\pi}{4} - 2.1\theta\right), (10^{-7}\mathrm{N\cdot m}), \text{Braked phase } (0.05, \infty) \end{cases}. \quad (s5.5)$$

Propelling force of Uni-SoPro, according to our previous study, this force is an unsteady term, since the bi-characteristics of the hydrodynamic force can be regarded as a result of the velocity competition between the Uni-SoPro and the liquid. Here, according to the estimation of tension-induced, viscous force-induced and momentum-induced propelling. We fit an attenuated function to estimate the propelling force in the acceleration state:

$$F_p = 5.9 \times 0.69^{200\times t-2}(10^{-4}\mathrm{N\cdot m}), \quad (s5.6)$$

where *t* is the time.

Supplementary Note 6. Analytical model for the control of Uni-SoPros

There are rich operation windows for us to program the motion behaviors of Uni-SoPros, e.g., magnetic direction, strength and duration of magnetic field and so on. Herein, we established a mathematical model to describe the relationship between operation parameters and theoretical target trajectory. Specifically, we describe the target trajectory by the angle θ_{n} and the distance l_{n}. In the motion programming, the critical control parameters include the control cycle *T* (consisting

of triggering cycle T_t, decelerating cycle T_d and steering cycle T_s), magnetic field **B**, space angle θ and φ.

To further simplify the model, we fixed some of the control parameters. Specifically, the magnetic field **B** is fixed at 100 Gs, magnetic field angle φ is set to from 170 to 120 degrees for triggering, and from 10 to 60 degrees for decelerating depending on the size of the Uni-SoPros. Then, the model can be further simplified as describing the relationship between θ, T and θ_n, l_n, where θ controls the direction, T determines the distance.

For direction control, as evidenced in Fig. 2h, it is fast enough. We only need to satisfy Eq. S6.1,

$$\theta = \theta_{\mathrm{n}}. \tag{s6.1}$$

For distance control, we first established a velocity model for the single trigger process. The whole process can be divided into three stages: triggering, decelerating, and stationary swerving. The velocity model can be expressed as follows,

$$v_{\mathrm{f}}(t) = \begin{cases} v_{\mathrm{f1}}(t) = a_1 - e^{b_1(t-t_0)+\ln(a_1)} & t_0 \le t < t_1 \\ v_{\mathrm{f2}}(t) = v_{\mathrm{f1}}(t_1) \cdot e^{b_2(t-t_1)} & t_1 \le t < t_2 \\ v_{\mathrm{f3}}(t) = v_{\mathrm{f2}}(t_2) \cdot e^{b_3(t-t_2)} & t_2 \le t < t_3 \\ v_{\mathrm{f4}}(t) = v_{\mathrm{f3}}(t_3) \cdot e^{b_4(t-t_3)} & t_3 \le t < t_4 \end{cases}, \tag{s6.2}$$

where $v_{\mathrm{f1}}(t)$ is the velocity function of the acceleration process in the triggering state, $v_{\mathrm{f2}}(t)$ is the velocity function of the stable process in the triggering state, $v_{\mathrm{f3}}(t)$ is the velocity function in the decelerating state, $v_{\mathrm{f4}}(t)$ is the velocity function in the stationary swerving state, a_1 determines the peak velocity, b_1 determines the acceleration during the acceleration process, b_2~b_4 determines the attenuation of speed. The t_1~t_4 can be expressed as follows,

$$\begin{cases} t_1 = t_0 + T_{\mathrm{up}} \\ t_2 = t_0 + T_{\mathrm{t}} \\ t_3 = t_2 + T_{\mathrm{d}} \\ t_4 = t_3 + T_{\mathrm{s}} \end{cases}, \tag{s6.3}$$

where t_0 is the origin of time, T_{up} is the acceleration duration. If $t_2 < t_1$, it means that the control magnetic field changes direction before the Uni-SoPro reaches its stable time. Combining Eq. s6.2 and Eq. s6.3, the velocity function can be expressed as a function of t, T_{up}, T_t, T_d and T_s,

$$v_{\mathrm{f}}(t) = v_{\mathrm{f}}(t, T_{\mathrm{up}}, T_{\mathrm{t}}, T_{\mathrm{d}}, T_{\mathrm{s}}), \tag{s6.4}$$

where T_{up} is determined by the structural size of the Uni-SoPros and the water environment. Integrating Eq. s6.4 with t, the theoretical distance l_n (the length of the target trajectory) can be obtained as,

$$l_\mathrm{n} = \int_{t_0}^{t_4} v_\mathrm{f}(t,\ T_\mathrm{up},\ T_\mathrm{t},\ T_\mathrm{d},\ T_\mathrm{s})dt\,. \tag{s6.5}$$

Theoretically, T_t, T_d and T_s that satisfy Eq. S5.5 have infinite number of solutions, considering the limited stable response frequency, we fixed T_d and T_s as 300 ms. Experiments show that the acceleration time T_{up} of the Uni-SoPro with a size scale of 0.8 in deionized water is ~0.1s (with fibres) and ~0.05s (without fibres). Thus, the relationship is only related to t and T_t as follows,

$$l_\mathrm{n} = \int_{t_0}^{t_4} v_\mathrm{f}(t,\ T_\mathrm{t})dt\,. \tag{s6.6}$$

Based on Eq. s6.6, we can solve T_t by l_n, where n represents the number of triggers.

As we already know that the existence of fibres in the tail has a great influence on the kinetic performance of the Uni-SoPros (Fig. 2f). Therefore, the speed curve of the continuous triggering process is related to n. Considering the effect of the attenuation of NOP release on speed, we modify Eq. s6.4 as follows,

$$v_\mathrm{f}(n, t,\ T_\mathrm{t}) = k(n) \cdot v_\mathrm{f}(t,\ T_\mathrm{t}), \tag{s6.7}$$

where $k(n)$ is the correction coefficient function which can be expressed as follows,

$$k(n) = \begin{cases} e^{-\lambda(n-1)}, & \text{(w/o fibres)} \\ \kappa(n-1)+1, & \text{(w/ fibres)} \end{cases}, \tag{s6.8}$$

where λ and κ are correction attenuation coefficients without/with fibres respectively, particularly, when the fibres is inserted, κ is approximately equal to zero in fewer trigger cycles. As suggested by Eq. s6.8, it can be concluded that the relationship between t and distance l_n should satisfy the following relationship,

$$l_\mathrm{n} = \int v_\mathrm{f}(n, t,\ T_\mathrm{t})dt\,. \tag{s6.9}$$

We took the Uni-SoPro with a size scale of 0.8 in deionized water as an example to validate the model (Fig. 6c), the parameters of the model are shown in Supplementary Table 6. The results show that the maximum error of the model within ten trigger cycles is -18.36 mm (without fibres)

and -5.75 mm (with fibres), as shown in Fig. 6d. The error mainly comes from the drift speed v_{drift} (~12 mm/s) and the sudden change of the posture (red dotted circle in Fig. 6c) in the process of decelerating and steering, and the uncontrollable factors of the environment.

Supplementary Note 7. Running constraints in the labyrinth

There are 13 corners and 3 dynamic lighting lamps that need to cross, when passing through the labyrinth (210 mm×300 mm, Supplementary Fig 20). The size parameters of the labyrinth are shown in Supplementary Table 4.

According to the rules we set, to successfully pass through the labyrinth, the following technical conditions should be met,

$$\Delta u_{\mathrm{i}} \in \left(-\frac{w_{\mathrm{c}}}{2}, \frac{w_{\mathrm{c}}}{2}\right), \mathrm{i} = 2\sim5, 7\sim15, \quad \text{(s7.1)}$$

$$\begin{cases} \Delta u_{\mathrm{i}} < -\frac{w_{\mathrm{l}}}{2}, \mathrm{i} = 1 \\ \Delta u_{\mathrm{i}} > \frac{w_{\mathrm{l}}}{2}, \mathrm{i} = 6, 16 \end{cases}, \quad \text{(s7.2)}$$

where Δu_{i} represents the cumulative error, and its value is equal to the cumulative sum of the horizontal error δ_{h} and the vertical error δ_{v}, as shown in Eq. s7.3,

$$\Delta u_{\mathrm{i}} = \begin{cases} \sum \delta_{\mathrm{h}}, & \mathrm{i} = 1,2,4,6,7,9,11,13,15 \\ \sum \delta_{\mathrm{v}}, & \mathrm{i} = 3,5,8,10,12,14,16,17 \end{cases}, \quad \text{(s7.3)}$$

where i presents the number of steps of the Uni-SoPro, w_{c} (28 mm) is the window width that can pass through the corner, w_{l} (11 mm) is the width of the lamp bar.

For corners, when the Uni-SoPro passes through the corner, it needs to finish decelerating, steering, and restarting within the passing window (light red bar, Supplementary Fig 20c) right panel.

For dynamic lamps, the Uni-SoPro should stop before the lamp bar when the light is on, and restart when the light is off, left panel (Supplementary Fig 20c).

The distance from the center line of the window or lamp when the Uni-SoPro restart is the error of each step. The horizontal error δ_{h} and the vertical error δ_{v} are independent of each other,

we defined that the error direction as shown in Supplementary Fig 20d. In addition, for every step, it should be ensured that the cumulative error of each step should satisfy Eq. s7.1 and Eq. s7.2[b].

[b] Nomenclatures used in this work are in Supplementary Table 7.

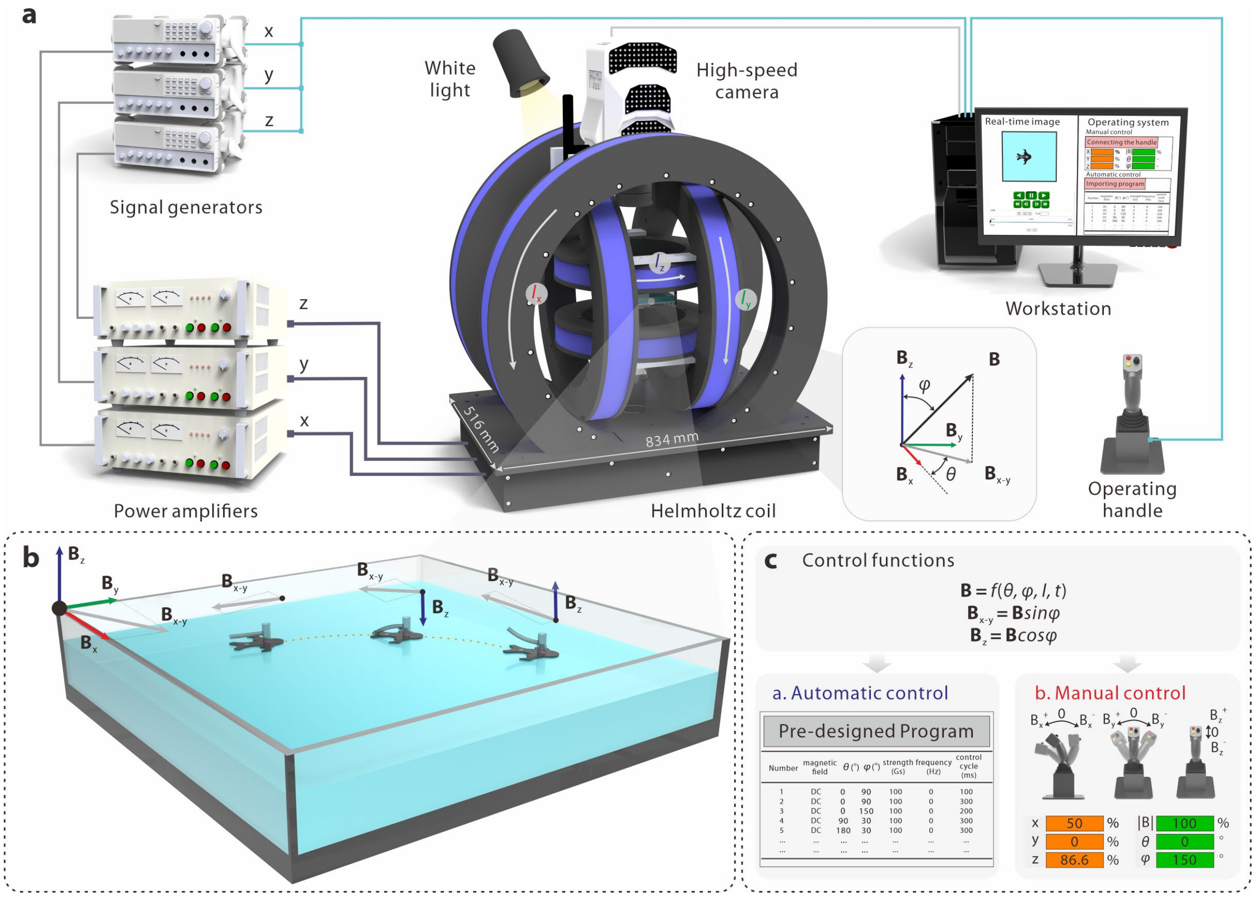

Supplementary Figure 1. Manipulation platform and operation schematics of Uni-SoPros. **(a)** A manipulation system consists of Helmholtz coils (up to 10 mT uniform magnetic field in the central spherical volume with a radius of 75 mm, uniformity ~95%), a high-speed imaging system, and an operation platform. All the kinematic characterizations were operated in such a magnetically uniform spherical area. **(b)** Schematic of Uni-SoPro actuation on water surface. **(c)** Two operational manners for manipulation of Uni-SoPros. Automatic control manner is used for pre-programmed actuation while manual control is used for real-time control.

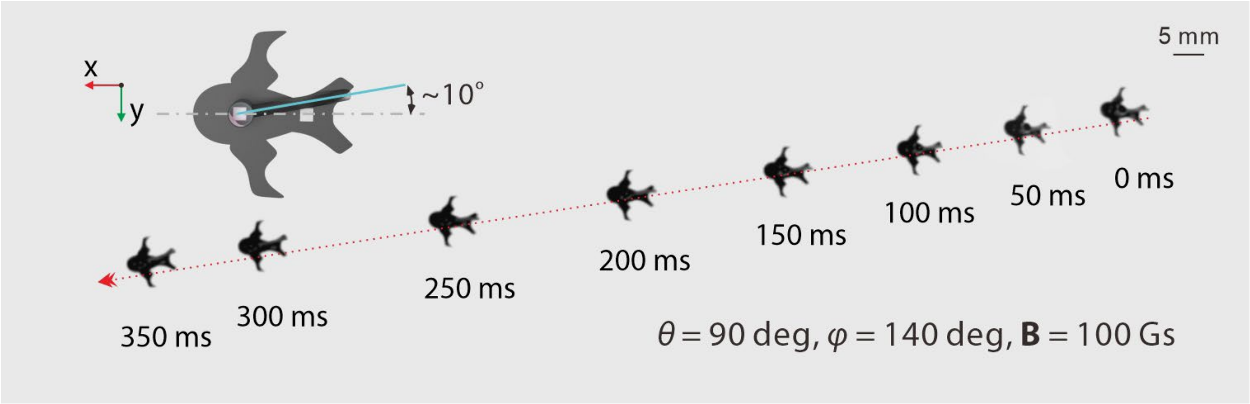

Supplementary Figure 2. Propulsion test with an off-center magnetic tail. The Uni-SoPro with an obliquely mounted magnetic tail (the angle between the magnetic tail and the main axis is ~10°) produces a diagonal trajectory under a normal manipulation magnetic fields (direction controlling magnetic field $\mathbf{B}_{x\text{-}y}$ is aligned with the ***x*** *axis*). The characteristic length of the Uni-SoPro in this test is 7.2 mm.

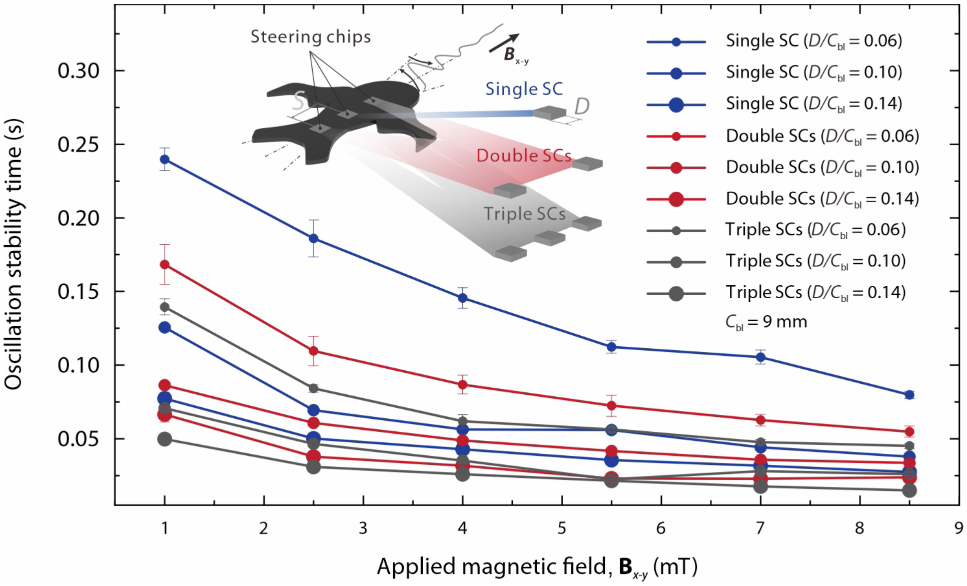

Supplementary Figure 3. Swerving characteristics. Effect of the applied magnetic field ($\mathbf{B}_{x\text{-}y}$) and the number and size of the SCs on the orientation oscillation stability time. Error bars indicate the standard deviation for n=3 sample measurements at each data point.

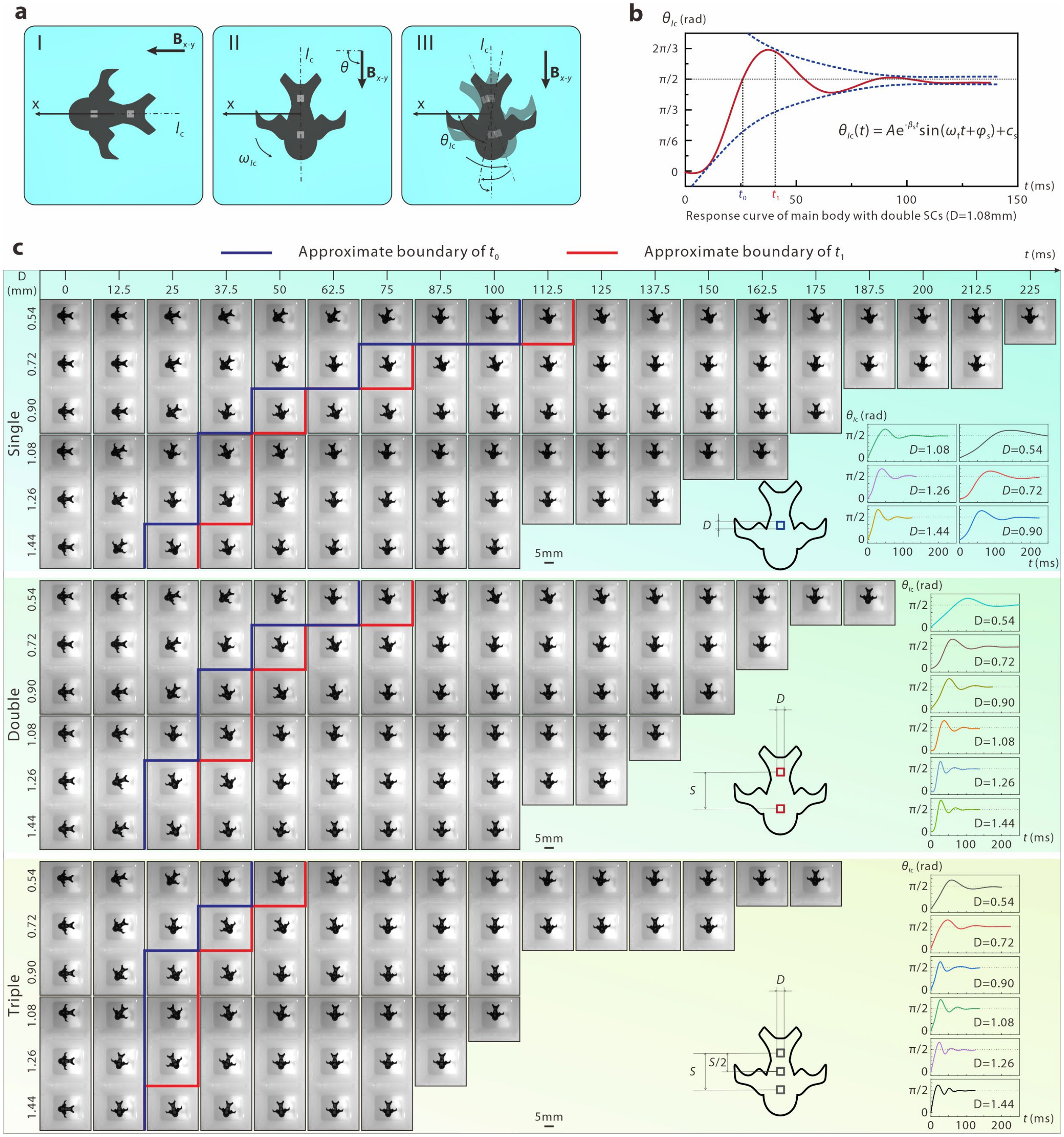

Supplementary Figure 4. Swerving characterization. (a) Schematics of the swerving process. **(b)** Underdamped vibration curve(*7*, *8*) of main body during swerving stabilization process, under the steering of a horizontal magnetic field. **(c)** Swerving oscillation processes of the main body with different numbers and sizes of SCs, under the same steering condition of 55 Gs horizontal magnetic field.

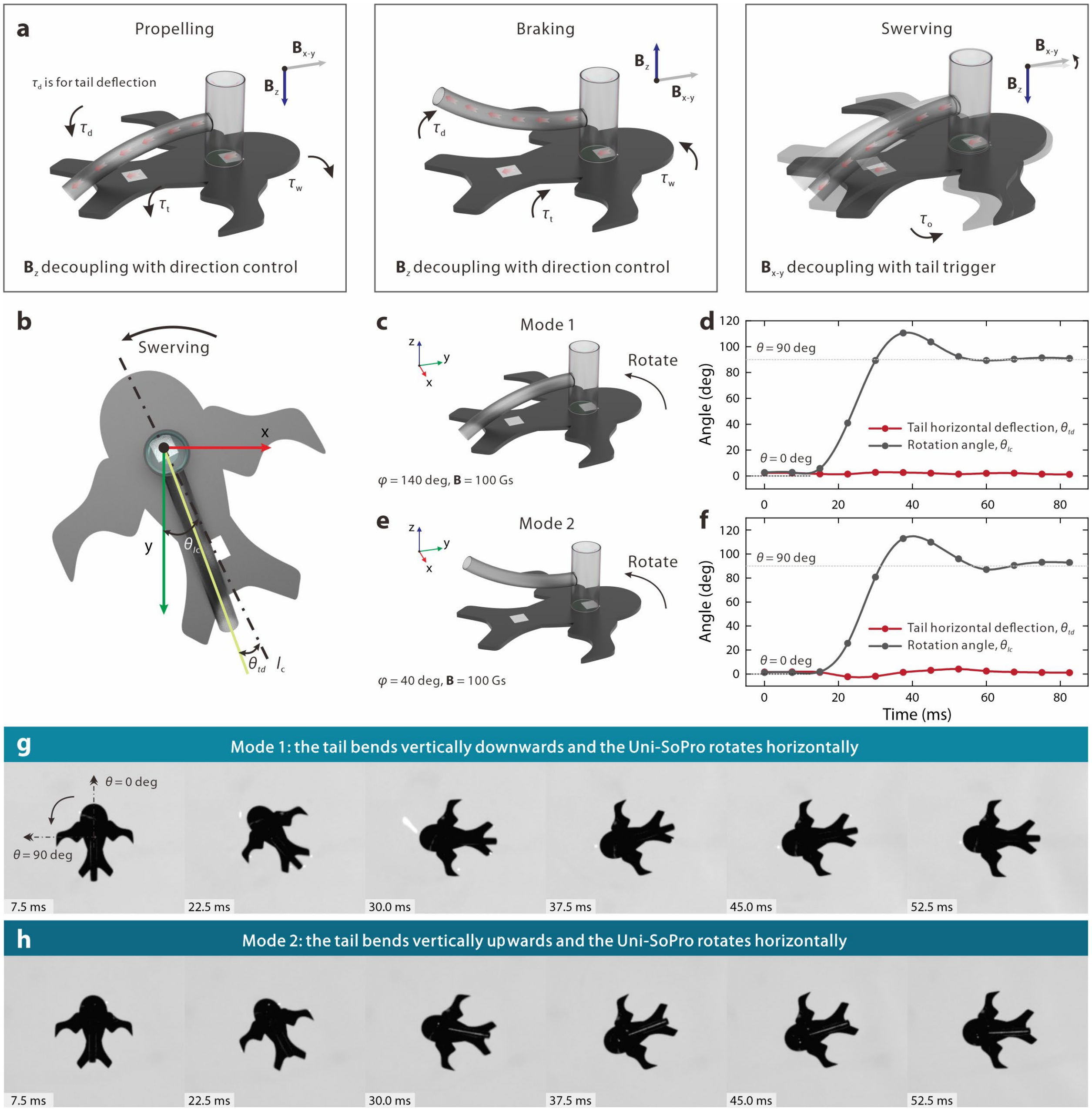
a
Propelling
τ_d is for tail deflection
$\mathbf{B}_{x\text{-}y}$
$\mathbf{B}_z$
τ_d
τ_t
τ_w
$\mathbf{B}_z$ decoupling with direction control
Braking
$\mathbf{B}_z$ decoupling with direction control
Swerving
τ_o
$\mathbf{B}_{x\text{-}y}$ decoupling with tail trigger
b
Swerving
x
y
θ_{lc}
θ_{td}
l_c
c
Mode 1
Rotate
φ = 140 deg, $\mathbf{B}$ = 100 Gs
d
θ = 90 deg
θ = 0 deg
Angle (deg)
Tail horizontal deflection, θ_{td}
Rotation angle, θ_{lc}
e
Mode 2
Rotate
φ = 40 deg, $\mathbf{B}$ = 100 Gs
f
Time (ms)
g
Mode 1: the tail bends vertically downwards and the Uni-SoPro rotates horizontally
θ = 0 deg
θ = 90 deg
7.5 ms
22.5 ms
30.0 ms
37.5 ms
45.0 ms
52.5 ms
h
Mode 2: the tail bends vertically upwards and the Uni-SoPro rotates horizontally
7.5 ms
22.5 ms
30.0 ms
37.5 ms
45.0 ms
52.5 ms

Supplementary Figure 5. Decoupled control. (a) Typical decoupled mechanism and typical actuation states. The magnetic tail deflection control is decoupled from Uni-SoPro's swerving as due to structural and magnetic domain presets. The horizontal component $\mathbf{B}_{x\text{-}y}$ ($\mathbf{B}\sin\varphi$) determines the direction of the Uni-SoPro, while the vertical component $\mathbf{B}_z$ ($\mathbf{B}\cos\varphi$) determines the bending/tilting degree of the tail. By controlling these two components, $\mathbf{B}_{x\text{-}y}$ and $\mathbf{B}_z$, respectively, Uni-SoPros can obtain determinant direction and propulsion state concurrently. **(b)** Schematic diagram illustrating the horizontal deflection of the tail which is concurrently deflected vertically either downwards or upwards, as the Uni-SoPro undergoes horizontal rotation. **(c-d)** Mode 1: The tail horizontal deflection when bent vertically downwards, and **(e-f)** Mode 2: The tail horizontal deflection when bent vertically upwards, both while the Uni-SoPro is rotated horizontally (from 0º to 90º). **(g-h)** High-speed camera keyframes (2000 fps) of decoupling motion of tail vertical deflection and Uni-SoPro's rotation. The characteristic length of Uni-SoPro is 7.2 mm in this experiment.

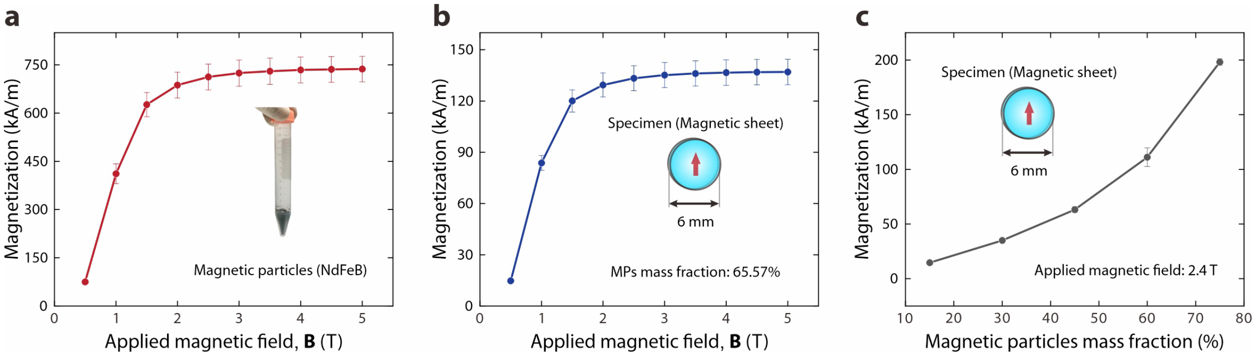

Supplementary Figure 6. Magnetization and response characterizations of magnetic particles and magnetic film. **(a)** Effect of applied magnetic fields on the magnetization of magnetic particles (MPs). **(b)** Effect of applied magnetic fields on the magnetization of magnetic films with 65.57% MPs mass fraction. **(c)** Effect of MPs mass fraction on the magnetization of magnetic films under an applied magnetic field of 2.4 T. Error bars indicate the standard deviation for *n*=3 sample measurements at each data point.

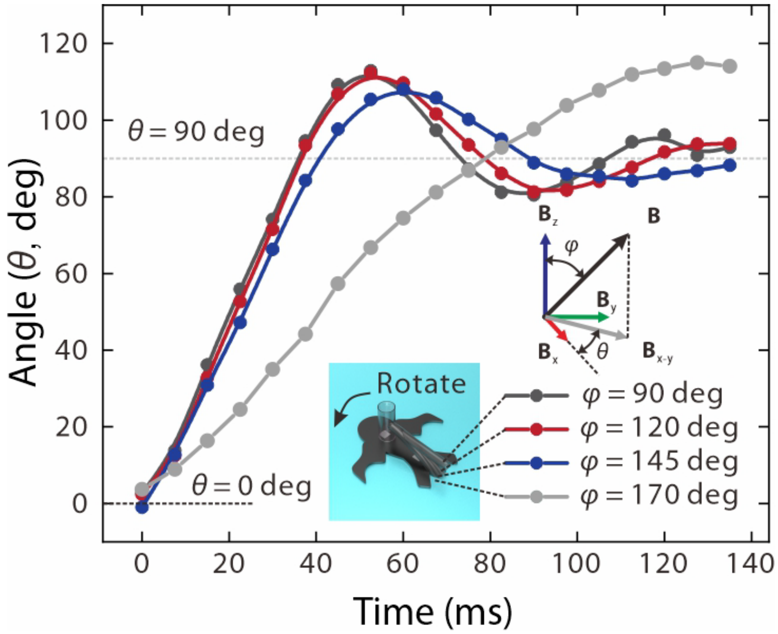

512 **Supplementary Figure 7. The influence of different postures and submerged magnetic tail**
513 **lengths on its swerving behaviour.** A Uni-SoPro with a size scale of 1.4 rotates under the
514 magnetic fields with different φ. The φ mainly affects the deflection of the magnetic tail and the
515 length of immersion in the water.

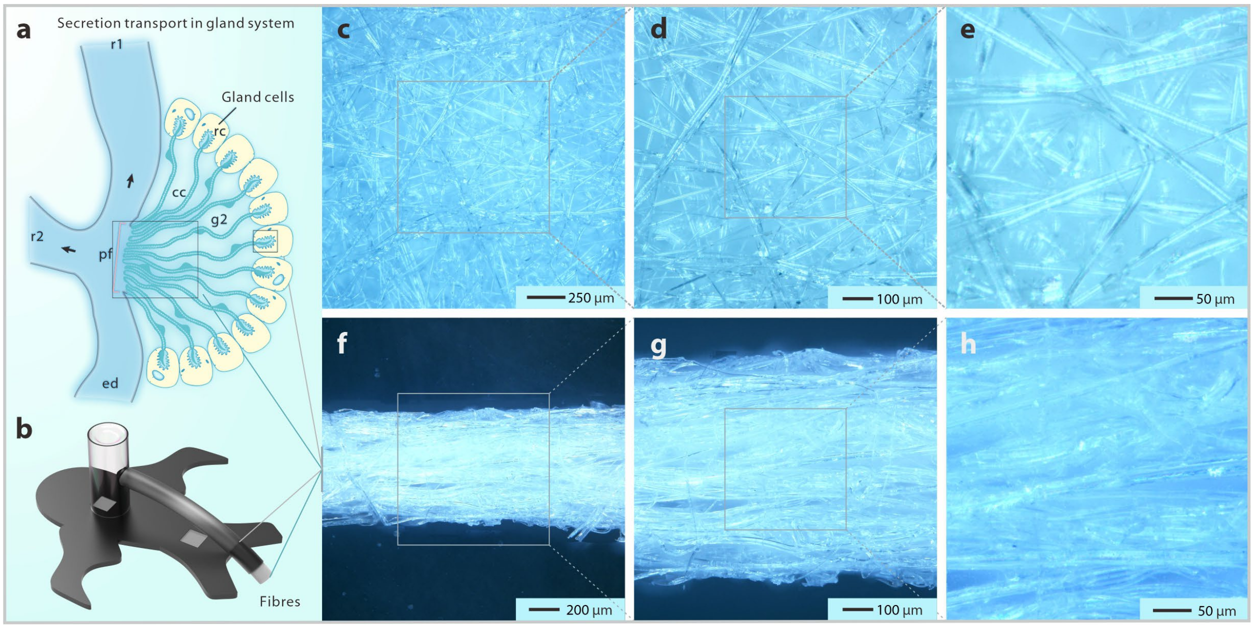

Supplementary Figure 8. Receiving/conducting canals-inspired inserted fibres at the tail tip of the Uni-SoPros. (a) Schematic drawings of the small gland system r2/g2 of *Stenus comma* (r2-small reservoir, g2-gland tissue associated to r2, r1-big reservoir, rc-receiving canal, cc-conducting canal, pf-pore field, ed-efferent duct of r1) (*2*). There are a bunch of micro canals for secretion transport from the gland cells to reservoirs. **(b)** The polypropylene (PP) fibres was analogously introduced and inserted at the tail tip of Uni-SoPros for fuel delivery optimization. **(c-e)** The 3D super depth of field reconstruction photos of the original PP fibres at different magnification views, where the diameter of fibres is ~15 µm (the diameter of receiving canal is ~1 µm). **(f-h)** The 3D super depth of field reconstruction photos of a bunch of inserted PP fibres of Uni-SoPro with a size scale of 1.2 at different magnification views. The fibres are twisted into a bundle to fit the tails, where most fibres are aligned along their axial direction while there are still some fibres arranged randomly.

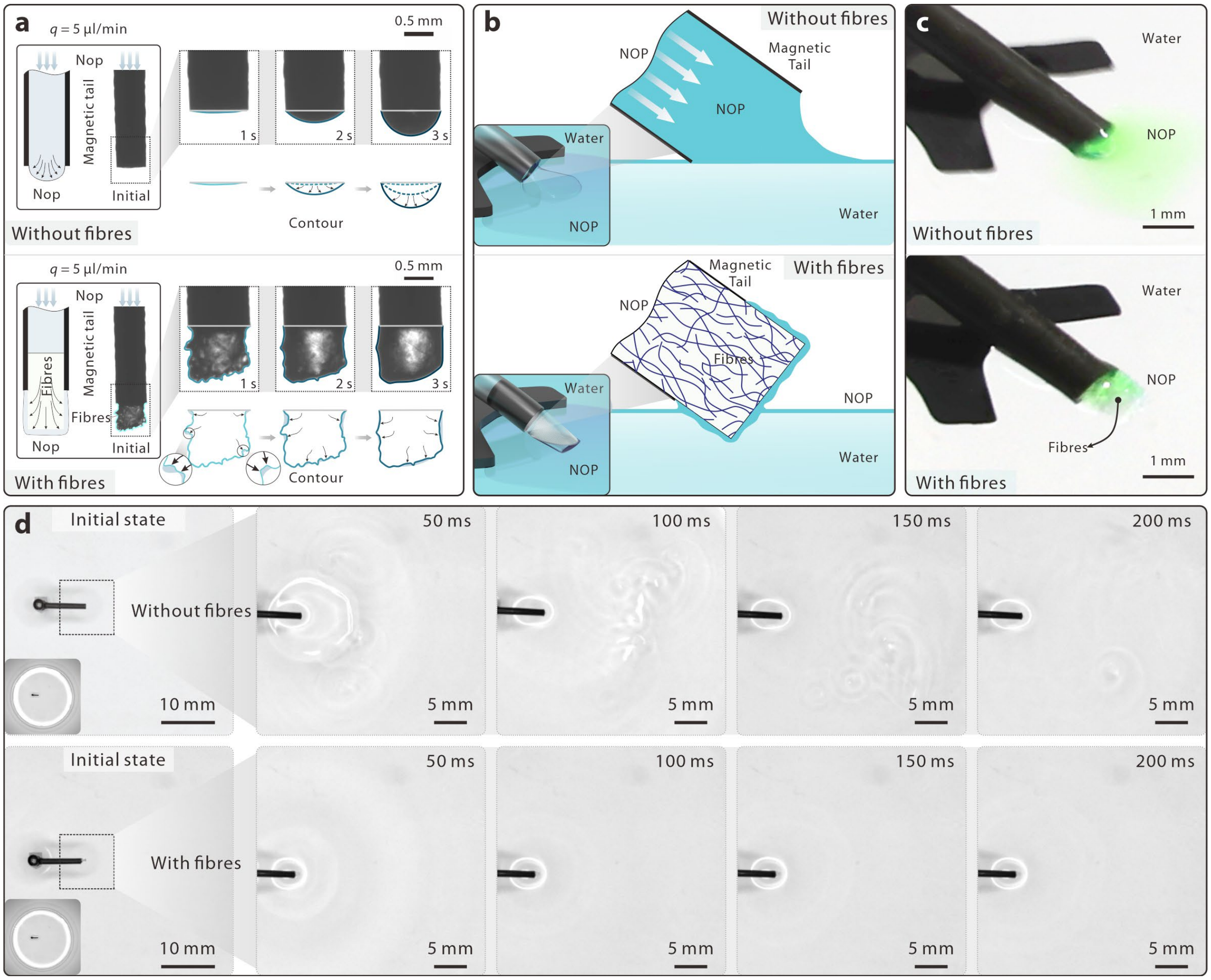

Supplementary Figure 9. Fuel (surfactant, NOP) release comparison of magnetic tail with/without inserted fibres. (a) Fuel release comparison at a constant flow rate (q=5 µl/min). The fuel flows out in a droplet state from the tail without inserted fibres, while the fuel flows out in a wetting thin layer from the tail with inserted fibres. **(b)** Schematics of releasing surfactant from two magnetic tails, where fibres inserted magnetic tail can smooth the fuel delivery process. **(c)** Visualized distribution of surfactant released on water surface, by deflecting magnetic tail touching the water surface for ~100 ms. The surfactant was dyed with pea green, the tail without fibres releases fuel faster than the one with fibres does under the same trigger condition. **(d)** High-speed images during fuel releasement. The fibres in the tail can serve as a physical buffer and homogenizer that makes the release process more linear and gentler. Here show the trigger and fuel spread process (Marangoni flow) of the magnetic tail with/without inserted fibres, respectively.

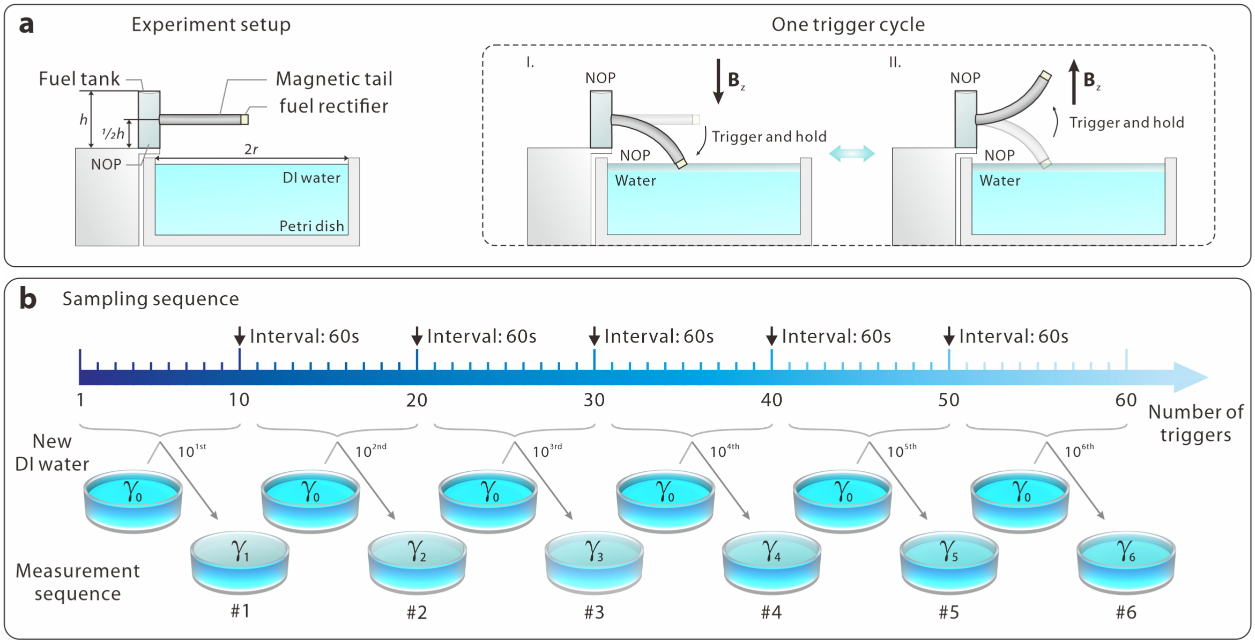

Supplementary Figure 10. Characterization of local surface tension tuning capability of magnetic tails with/without inserted fibres. (a) Experimental setup and fuel delivery cycle schematic. **(b)** DI water renewing sequence, trigger sequence and measurement sequence in the surface tension tuning test. The water surface radius in this test is 2.85 cm and whole DI water in Petri dish will be renewed after every ten triggers.

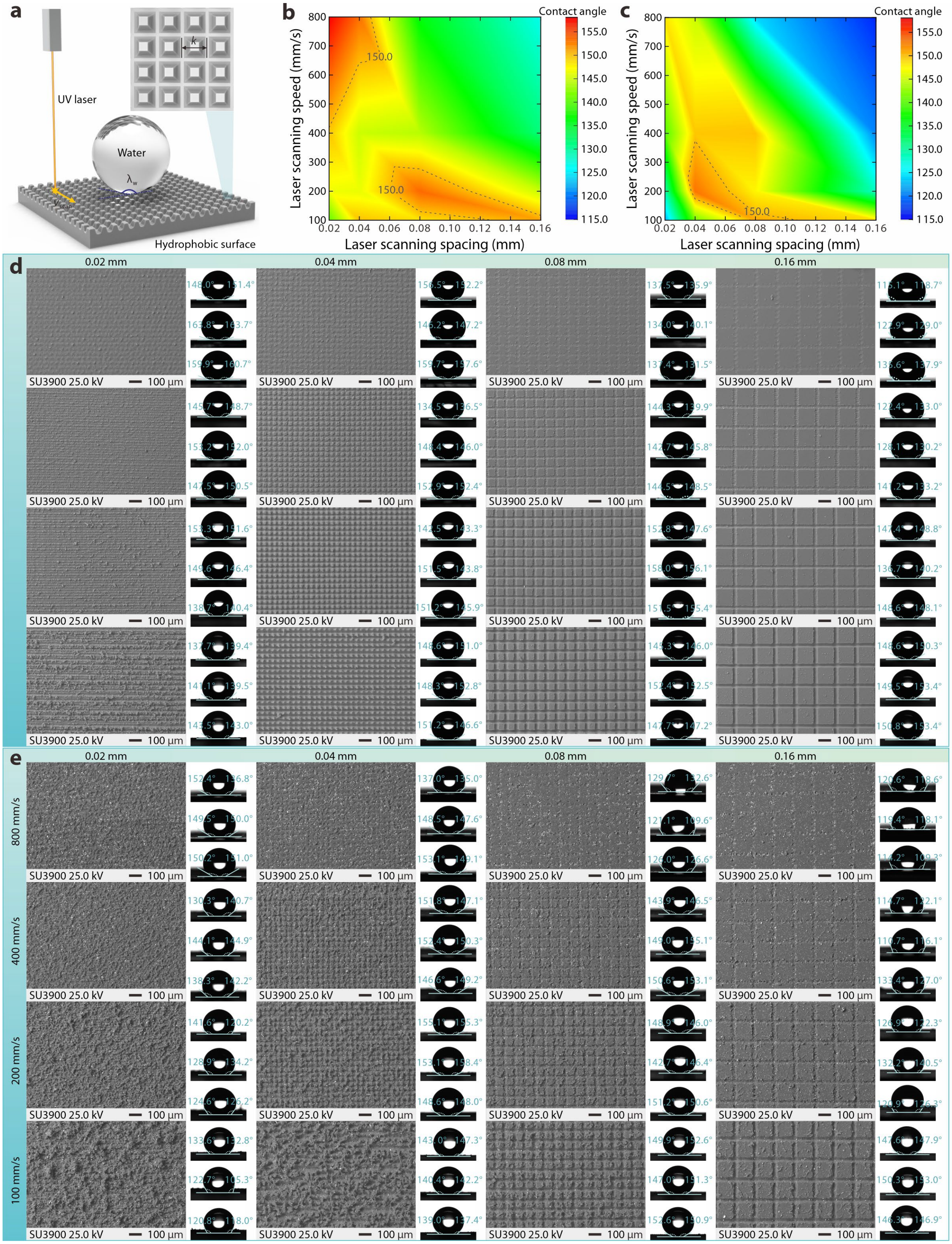
a
UV laser
Water
λ_w
k
Hydrophobic surface
b
c
Contact angle
155.0
150.0
145.0
140.0
135.0
130.0
125.0
120.0
115.0
Laser scanning speed (mm/s)
Laser scanning spacing (mm)
0.02
0.04
0.06
0.08
0.10
0.12
0.14
0.16
100
200
300
400
500
600
700
800
d
e
0.02 mm
0.04 mm
0.08 mm
0.16 mm
800 mm/s
400 mm/s
200 mm/s
100 mm/s
SU3900 25.0 kV
100 µm

Supplementary Figure 11. Surface hydrophobization treatment and characterizations. (a) Schematic of surface hydrophobization treatment via programmed UV laser scanning. **(b, c)** Static contact angle mapping of c-PDMS and m-PDMS films under various laser scanning spacing and scanning speeds, respectively. **(d, e)** Scanning electron microscope (SEM) images of the surface morphology and contact angle images of c-PDMS and m-PDMS films under various treatment parameters.

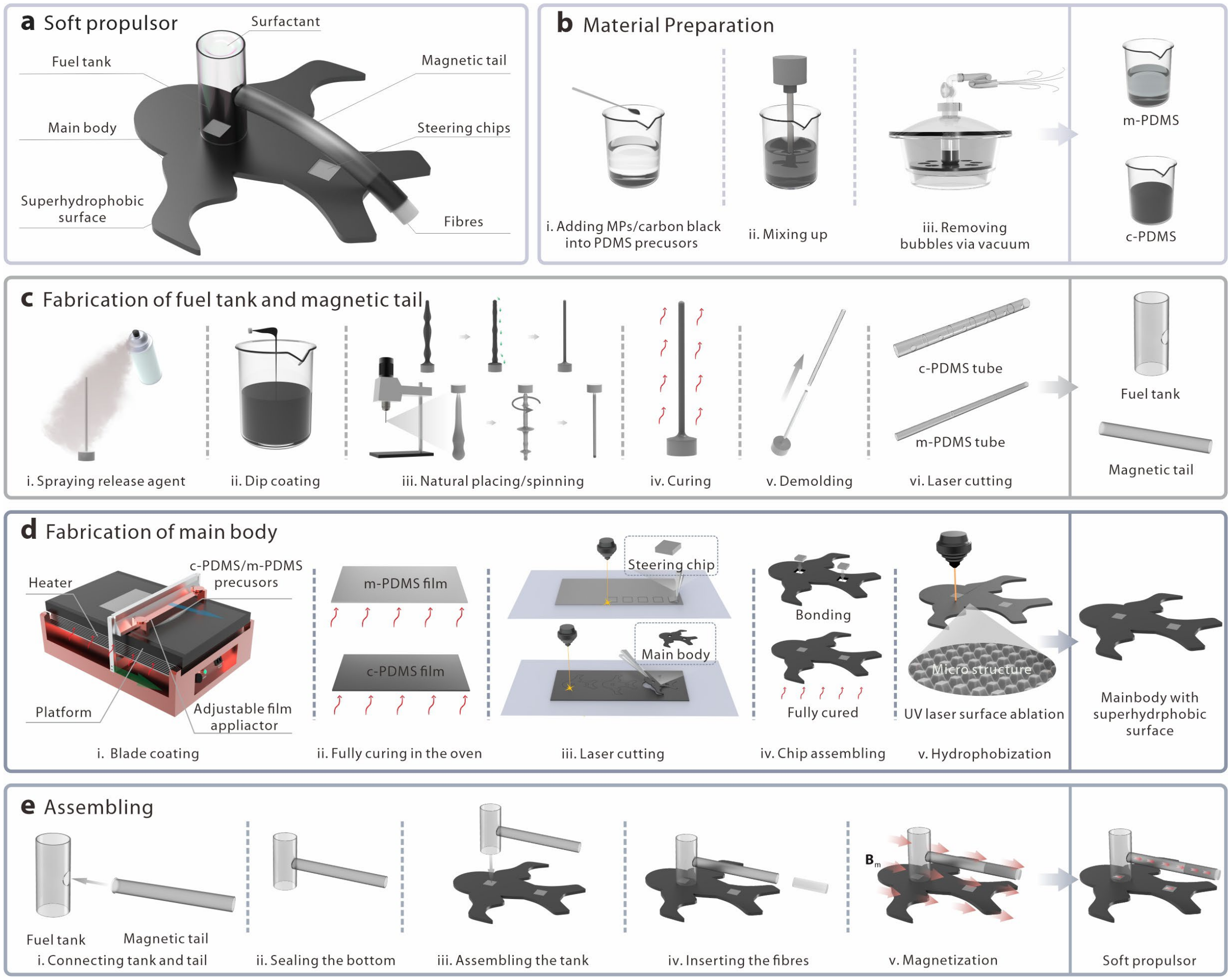

Supplementary Figure 12. Fabrication processes. (a) Main components of a Uni-SoPro. **(b)** c-PDMS and m-PDMS precursor mixture preparation. **(c)** Fabrication of fuel tank and magnetic tail. **(d)** Fabrication of the main body with SCs. **(e)** Assembly, fibres insertion, magnetization, and fuel loading.

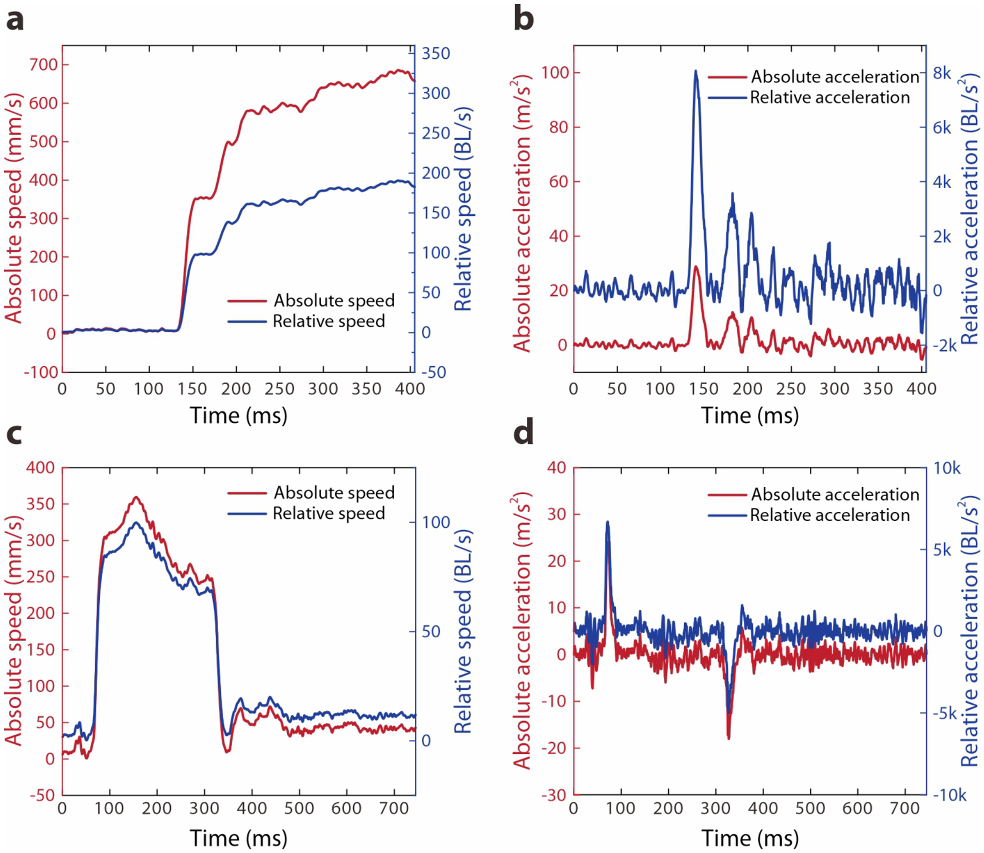

Supplementary Figure 13. Detailed velocity and acceleration curves of Uni-SoPros in the scale effect characterization and braking test. (a, b) Velocity and acceleration curves of a Uni-SoPro with a characteristic length of 3.6 mm in the scale effect characterizations, showing a peak relative speed of ~201 BL/s (725 mm/s) and peak relative acceleration of ~8,372 BL/s^2 (30 m/s^2) in the initial acceleration state. **(c, d)** Velocity and acceleration curves of a Uni-SoPro with a characteristic length of 3.6 mm in the braking test, show a deceleration of ~-5,010 BL/s^2 (18 m/s^2).

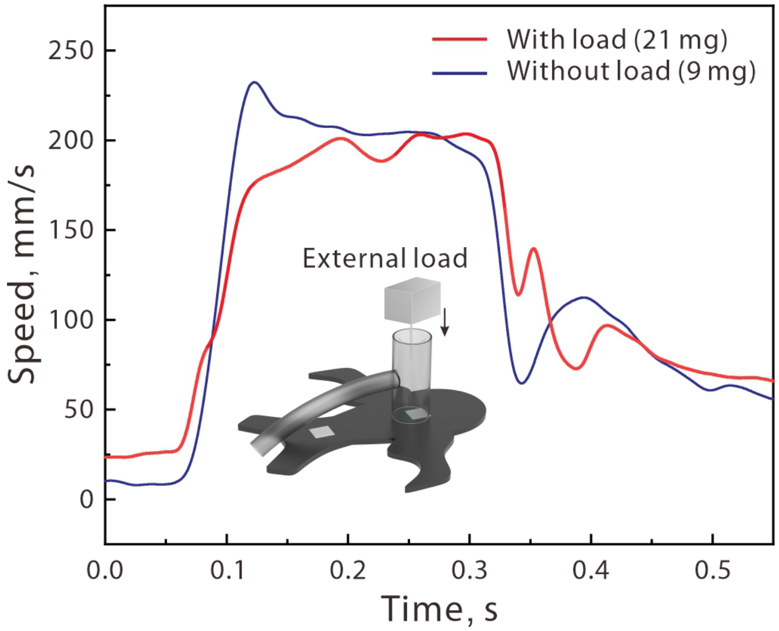

561 **Supplementary Figure 14. Loading ability characterizations of Uni-SoPros.** The Uni-SoPro
562 loading 12 mg cargo shows a slight kinematic attenuation compared with the one that without load.

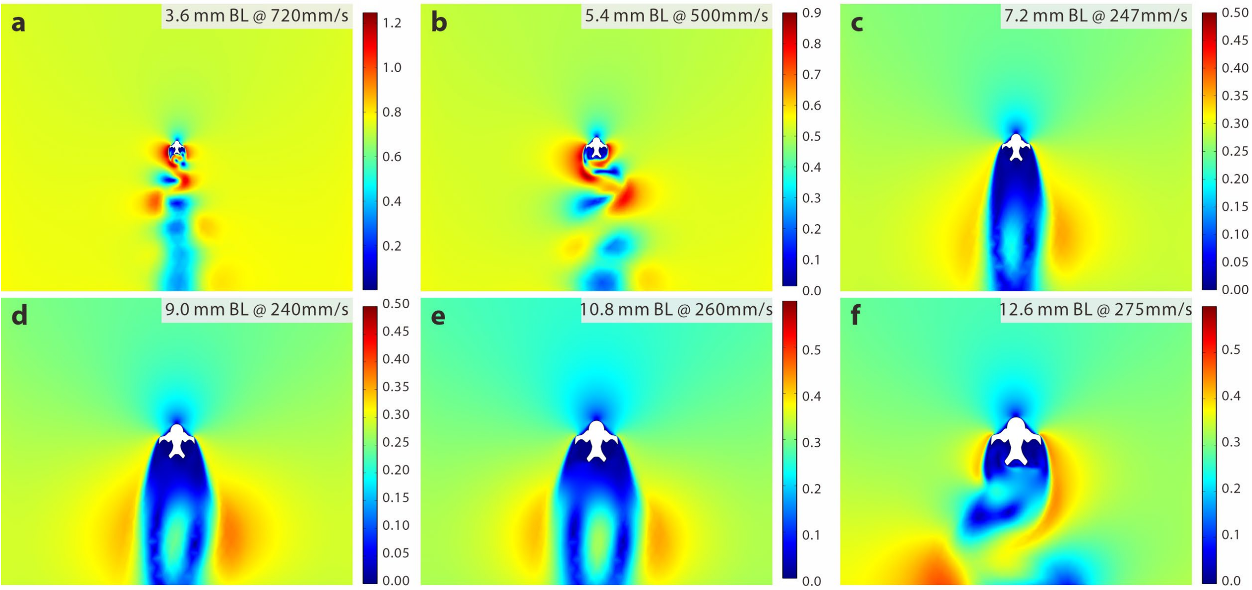

Supplementary Figure 15. Simulated flow velocity mapping of each size of Uni-SoPros at its peak speed with a horizontal posture. (a-f) COMSOL-simulated flow velocity distribution around the Uni-SoPros with characteristic lengths from 3.6 mm to 12.6 mm at their corresponding peak speeds.

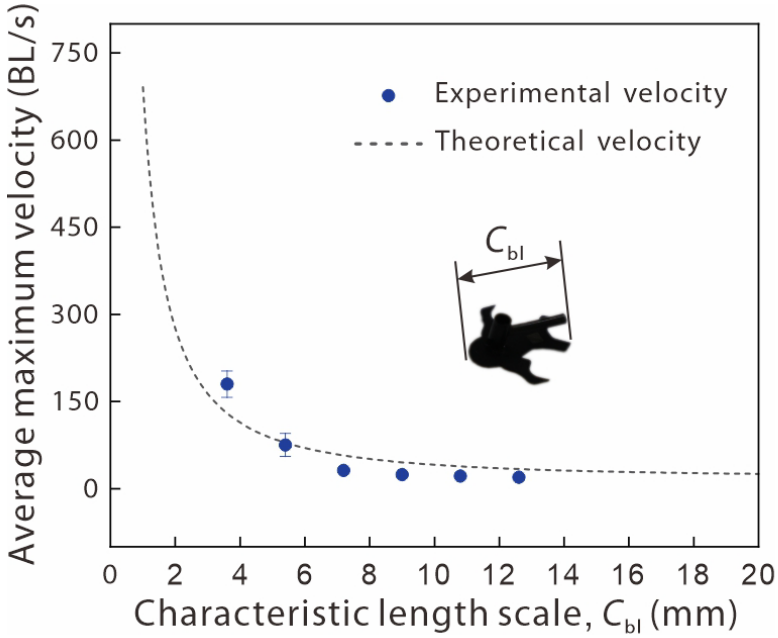

Supplementary Figure 16. Scaling analysis. The relative peak speed is a function of the characteristic length C_{bl}. Error bar represents standard deviation, n=3.

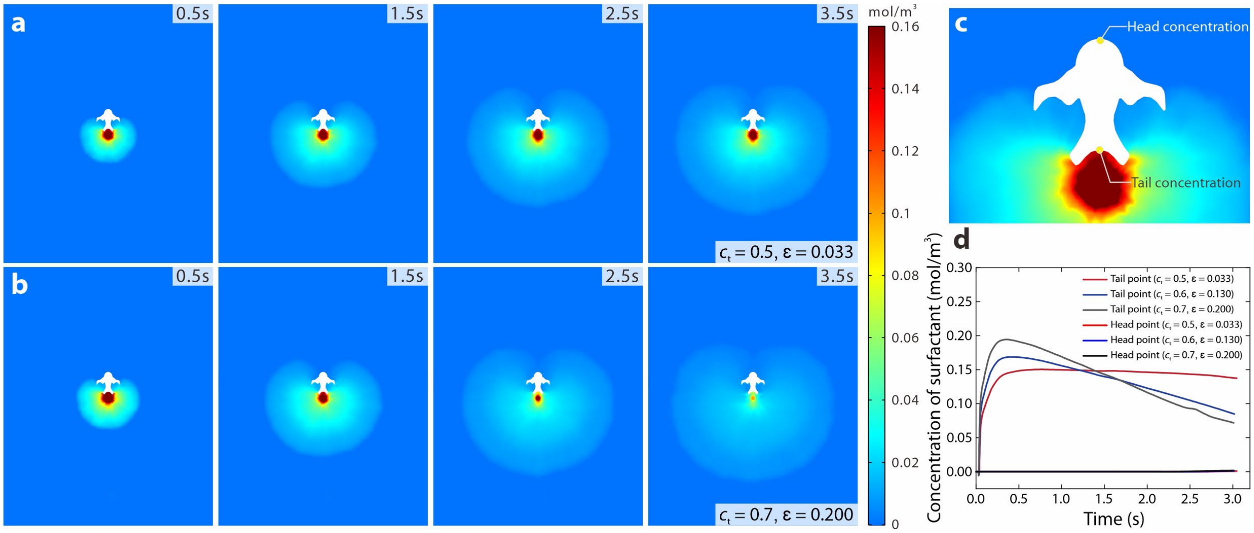

Supplementary Figure 17. Comparison of surfactant release with different strategies via numerical simulation. (a, b) Surfactant concentration mapping during spread process with different delivery functions. **(c)** Schematic of the position of head point (0, 200 mm) and tail point (0, 181 mm) at the contacting line. **(d)** Concentration variation of surfactants at the head point and tail point under various surfactant delivery functions. The fuel release with low attenuation coefficient shows a steadier concentration gradient than that of the degressive one (with a relatively larger attenuation coefficient).

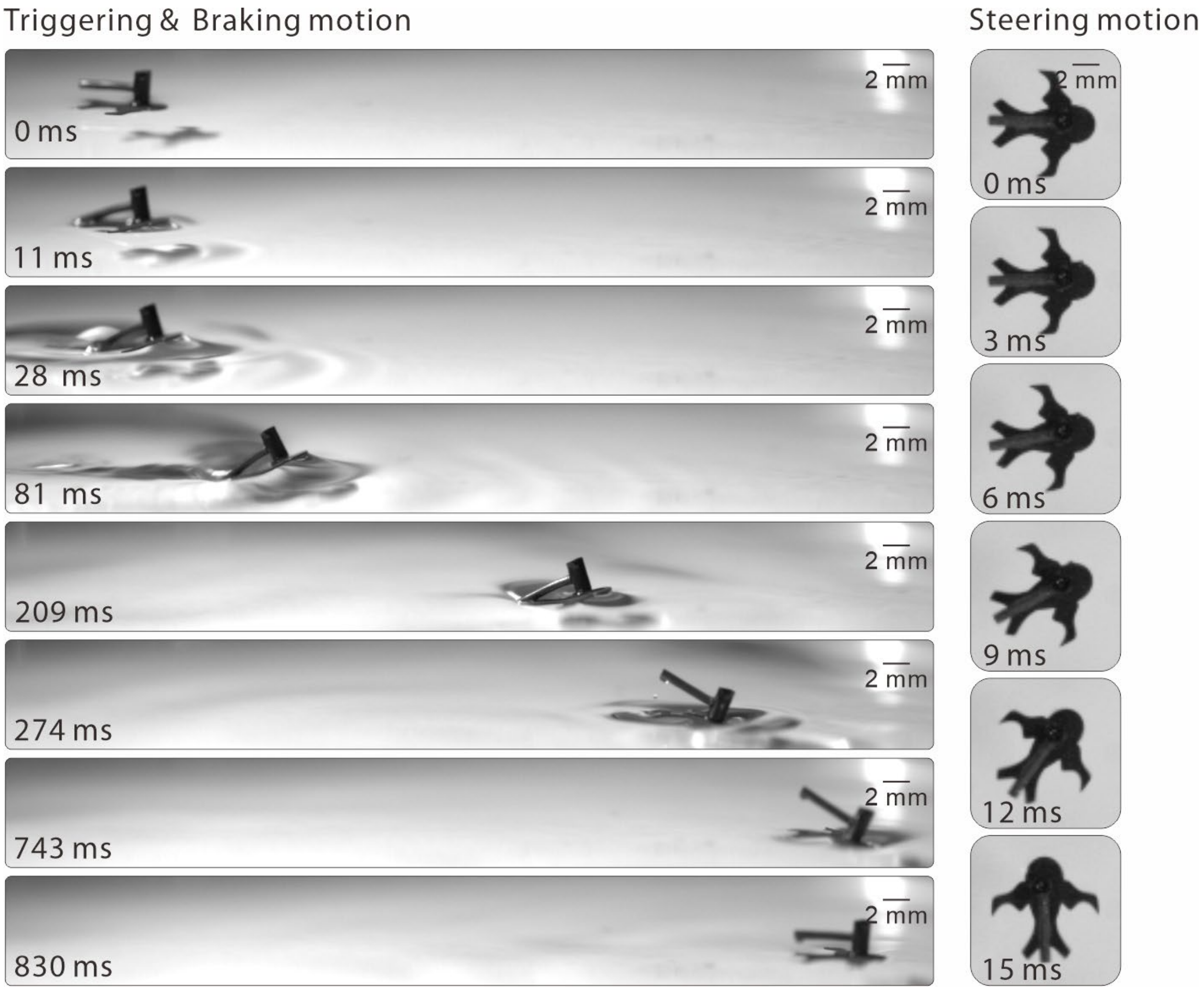

576 **Supplementary Figure 18. Snapshots of triggering, braking, and steering motion extracted**
577 **from the high-speed recoding system.**

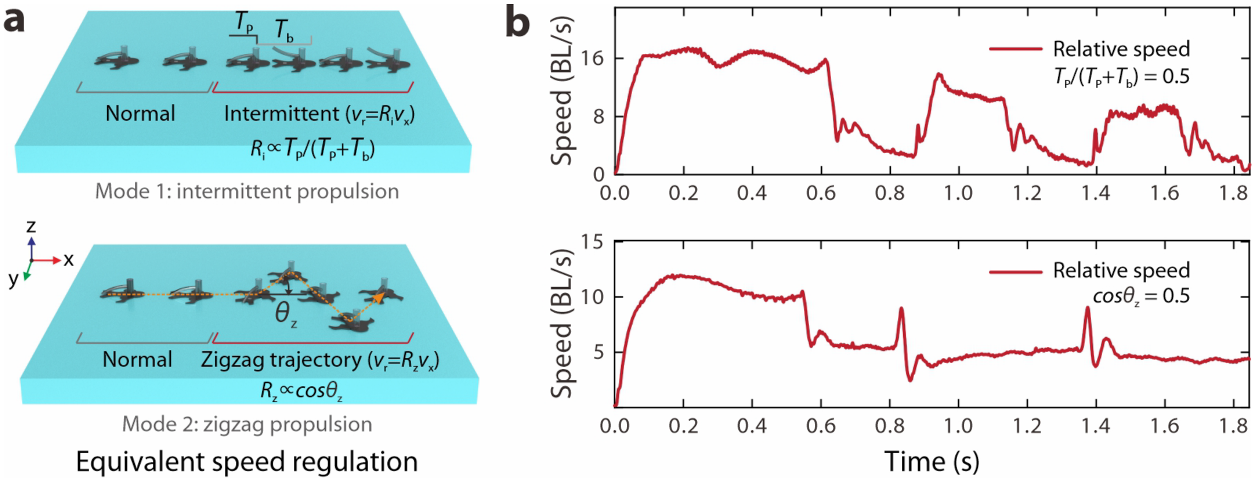

Supplementary Figure 19. Two equivalent speed regulation strategies. (a) Schematic depicting the concept concepts of intermittent propulsion (mode 1) and zigzag propulsion (mode 2). In mode 1, speed control is achieved by manipulating the speed attenuation coefficient R_i, which is proportionally related to $T_p/(T_p+T_b)$. In mode 2, speed regulation relies on the speed attenuation coefficient R_z, which exhibits a positive correlation with $\cos\theta_z$. **(b)** The speed regulation for a Uni-SoPro with a scale of 1.2 in intermittent propulsion ($T_p/(T_p+T_b) = 0.5$) and in zigzag propulsion ($\cos\theta_z = 0.5$).

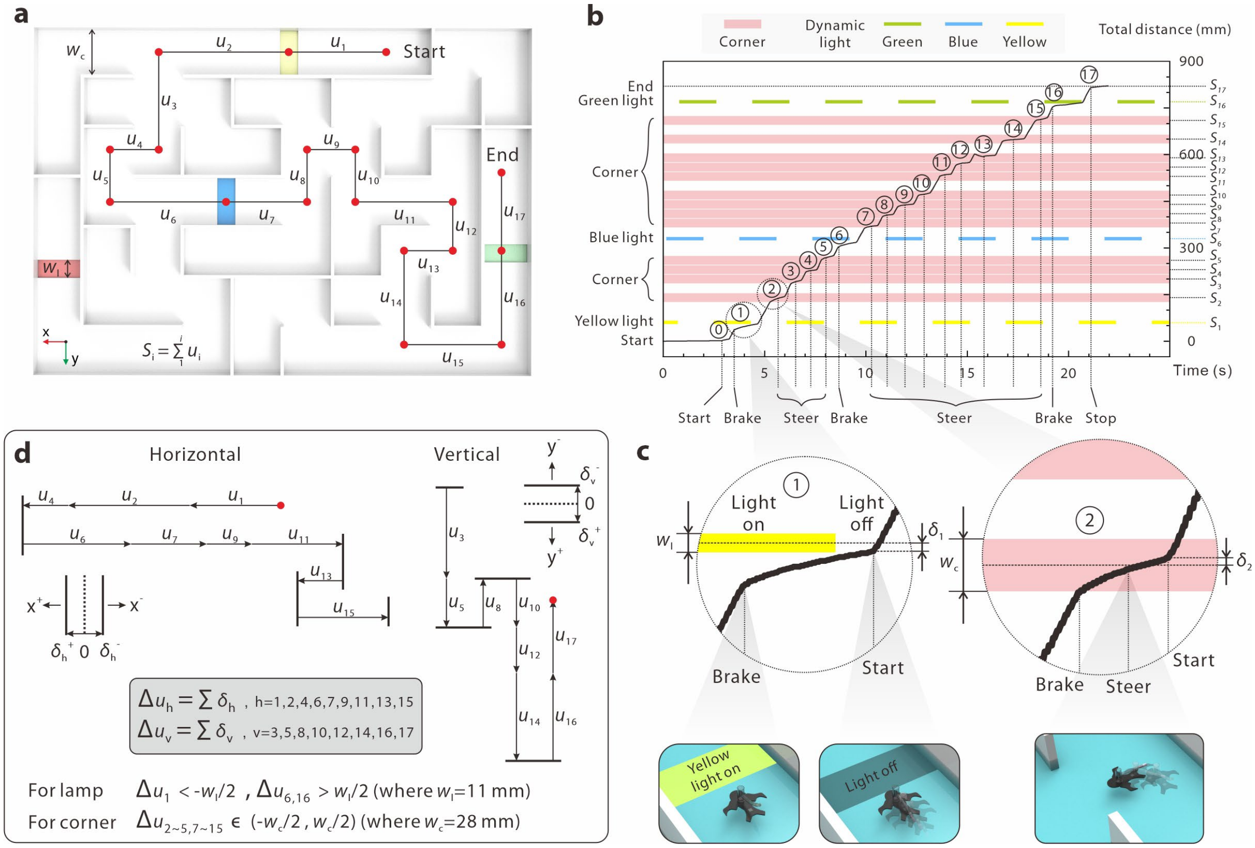

Supplementary Figure 20. Design and given constraints of the labyrinth with dynamic lights. **(a)** Critical design parameters of the labyrinth. **(b)** Action windows for the Uni-SoPro from the start point to the end point. **(c)** Schematics of a typical lamp obstacle and turning corner. **(d)** Accumulated displacement error constraints during the whole process.

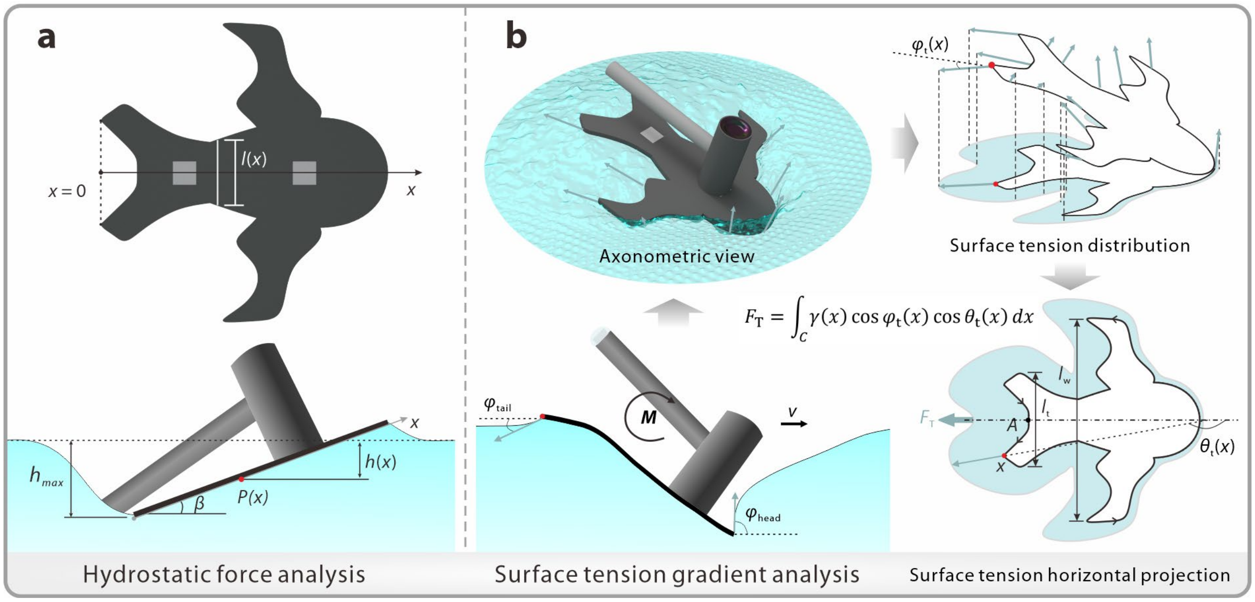

Supplementary Figure 21. Illustration of hydrostatic forces and surface tension difference analysis. (a) Axis x starts from the tail point. When α and maximum depth h_{max} are measured, the force can be calculated in MATLAB, where l(x) has been an array of 1547 columns. **(b)** Axis x starts from the tail point. Tension at x is a vector. Its amplitude is γ_1 or γ_0. Two angles $\varphi_t(x)$ and $\theta_t(x)$ describe the direction of the tension at x.

Supplementary Tables

Supplementary Table 1. Comparison of comprehensive kinematic performance.

Category	Subclass	Body length (m)	Peak velocity (m/s)	Peak acceleration (m/s^2)	Peak relative velocity (BL/s)	Peak relative acceleration (BL/s^2)	CKPI (BL^2/s^3)
This work	Uni-SoPro	0.0036	~0.72	~30	**~200**	**~8000**	**~1.6×10^{6}**
Tension-induced swimmers	Micro swimmers (*9*)	0.012	0.05	0.19	4.17	**15.83**	**6.6×10^{1}**
	Untethered fully soft robot (*10*)	0.045	0.24	0.29	**5.50**	6.60	3.6×10^{1}
Mammal	Cheetah (*11*)	1.0500	**19.900**	**8.300**	**18.95**	**7.90**	**1.5×10^{2}**
	Impala (*11*)	1.3200	13.800	5.700	10.45	4.32	4.5×10^{1}
	Lion (*11*)	1.7600	13.900	5.200	7.90	2.95	2.3×10^{1}
	Zebra (*11*)	2.1400	10.600	3.900	4.95	1.82	9.0×10^{0}
	Rats (*12*)	0.2000	1.250	1.030	6.25	5.15	3.2×10^{1}
	Bolt (*13*)	1.9500	12.340	5.659	6.33	2.90	1.8×10^{1}
Arthropods	Dragonfly (*14*)	0.0557	**3.400**	25.000	61.04	448.83	2.7×10^{4}
	Flat spider (*15*)	0.0095	0.600	43.600	63.16	**4589.47**	2.9×10^{5}
	Grasshopper (*16*)	0.0750	3.210	**220.960**	42.80	2946.13	1.3×10^{5}
	Mosquito (*17*)	0.0040	0.400	17.590	**100.00**	4397.50	**4.4×10^{5}**
	Water strider (*18*)	0.0118	0.991	11.773	84.00	997.71	8.4×10^{4}
Fish	Bass (*19*)	0.2360	2.500	110.000	10.59	466.10	4.9×10^{3}
	Goldfish (*20*)	0.0850	**2.899**	247.735	34.10	2914.53	9.9×10^{4}
	Catfish (*20*)	0.0920	2.714	**271.400**	29.50	2950.00	8.7×10^{4}
	Garfish (*20*)	0.1200	2.364	189.120	19.70	1576.00	3.1×10^{4}
	Common hatchetfish (*20*)	0.0210	1.407	140.700	**67.00**	**6700.00**	**4.5×10^{5}**
	Spiny eel (*20*)	0.2550	2.175	185.910	8.53	729.06	6.2×10^{3}
Soft robots	GoQBot (*21*)	0.1000	0.750	73.397	7.50	733.97	5.5×10^{4}
	Fish robot (*22*)	0.3400	0.190	0.220	0.56	0.65	3.6×10^{-1}
	Small legged robot (*23*)	0.0700	0.183	1.970	2.61	28.14	7.3×10^{1}
	Explosion jump robot (*24*)	0.0800	**3.430**	**114.33**	**42.88**	**1429.13**	**6.1×10^{4}**
	Wormlike robot (*25*)	0.2000	0.041	0.296	0.21	1.48	3.1×10^{-1}
Rigid robots	1STAR (*26*)	0.1200	0.350	3.920	2.92	32.67	9.5×10^{1}
	DC motor (*27*)	0.1040	**4.900**	28.170	47.12	270.87	1.3×10^{4}
	Sprawlita (*28*)	0.1600	0.625	8.580	3.91	53.63	2.1×10^{2}
	Climbing robot (*29*)	0.4000	0.560	5.400	1.40	13.50	1.9×10^{1}
	Jumping strider (*30*)	0.0980	1.670	**207.783**	17.04	2120.23	3.6×10^{4}
	Locust-inspired robot (*31*)	0.14	9	400	**64.29**	**2857.14**	**1.8×10^{5}**

Comprehensive kinematic performance index (CKPI, BL^2/s^3=peak relative acceleration, BL/s^2 × peak relative velocity, BL/s) is introduced to simultaneously evaluate the two-dimensional performance (relative velocity and relative acceleration) of the natural livings and robots. Some kinematic performance values are estimated or calculated from figures, data or videos reported.

Supplementary Table 2. Design and fabrication parameters of various Uni-SoPros.

**Design and Fabrication Parameters**			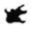	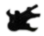	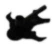	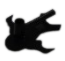	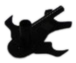	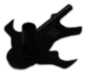
		Size scale (Absolute BL/$C_{bl1.0}$)	0.4	0.6	0.8	1.0	1.2	1.4
Body		Length (mm)	3.45~3.60	5.17~5.40	6.90~7.20	8.62~9.00	10.34~10.80	12.07~12.60
Body		Width (mm)	3.59	5.39	7.18	8.98	10.77	12.57
Body		Thickness (μm)	300	300	300	300	300	300
Body		Size of magnetic chip (mm)	0.43×0.43	0.65×0.65	0.86×0.86	1.08×1.08	1.30×1.30	1.51×1.51
Body		Spacing of magnetic chip (mm)	1.44	2.16	2.88	3.6	4.32	5.04
Body	Laser Surface Treatment	Speed (mm/s)	800	800	800	800	800	800
Body	Laser Surface Treatment	Spacing (mm)	0.02	0.02	0.02	0.02	0.02	0.02
Body	Laser Surface Treatment	Scan times (Top)	2	2	2	2	2	2
Body	Laser Surface Treatment	Scan times (Bot)	2	2	2	2	2	2
Tube		Length (mm)	2.2	3.3	4.4	5.5	6.6	7.7
Tube		Caliber of tube (mm)	0.24	0.36	0.48	0.6	0.72	0.84
Tube		Speed of spin coating (RPM)	19k~22k	11k~14k	5k~7k	N/A	N/A	N/A
Tank		Height of tank (mm)	1.2	1.8	2.4	3.0	3.6	4.2
Tank		Caliber of tank (mm)	0.5	0.75	1.0	1.25	1.5	1.75
Tank		Speed of spin coating (RPM)	11k~14k	8k~10k	5k~7k	N/A	N/A	N/A

The size scale of the Uni-SoPros was defined as the ratio of the absolute body length to 9 mm.

Supplementary Table 3. Manipulation magnetic field codes for characterizations.

Term		Number	θ (°)	φ (°)	Strength (Gs)	Control cycle (ms)	Number	θ (°)	φ (°)	Strength (Gs)	Control cycle (ms)
Swevring test	N/A	1	90	90	10	1500	7	90	90	55	1500
		2	0	90	95	1000	8	0	90	95	1000
		3	90	90	20	1500	9	90	90	70	1500
		4	0	90	95	1000	10	0	90	95	1000
		5	90	90	40	1500	11	90	90	85	1500
		6	0	90	95	1000	12	0	90	95	1000
Performance measurement for different sized Uni-SoPros	0.4	1	0	90	100	1500	3	0	10	100	300
		2	0	170	100	200	4	0	20	100	1000
	0.6	1	0	90	100	1500	3	0	20	100	300
		2	0	160	100	200	4	0	20	100	1000
	0.8	1	0	90	100	1500	3	0	40	100	300
		2	0	140	100	200	4	0	20	100	1000
	1.0	1	0	90	100	1500	3	0	50	100	300
		2	0	130	100	200	4	0	20	100	1000
	1.2	1	0	90	100	1500	3	0	60	100	300
		2	0	120	100	200	4	0	20	100	1000
	1.4	1	0	90	100	1500	3	0	60	100	300
		2	0	120	100	200	4	0	20	100	100
Surface tension measurement	200/600/1000 ms	1	90	0	100	200/600/1000	11	90	0	100	200/600/1000
		2	90	180	100	500	12	90	180	100	500
		3	90	0	100	200/600/1000	13	90	0	100	200/600/1000
		4	90	180	100	500	14	90	180	100	500
		5	90	0	100	200/600/1000	15	90	0	100	200/600/1000
		6	90	180	100	500	16	90	180	100	500
		7	90	0	100	200/600/1000	17	90	0	100	200/600/1000
		8	90	180	100	500	18	90	180	100	500
		9	90	0	100	200/600/1000	19	90	0	100	200/600/1000
		10	90	180	100	500	20	90	180	100	500

Number represents the magnetic field code sequence for manipulation of Uni-SoPros, and 0.4-1.4 represents the size scale of Uni-SoPros for performance characterizations. 200 ms, 600 ms, and 1000 ms represent the touching time with water. The magnetization direction of the magnetic tail in surface tension measurement is opposite with other characterization.

Supplementary Table 4. Critical running parameters in the labyrinth.

Step	Event	Motion	Spacing (mm)		Total distance (mm)	
0	Start	Triger	u_0	-	S_0	0
1	Yellow lamp	Brake	u_1	60	S_1	60
2	Corner	Turn left	u_2	80	S_2	140±14
3	Corner	Turn right	u_3	60	S_3	200±14
4	Corner	Turn left	u_4	30	S_4	230±14
5	Corner	Turn left	u_5	30	S_5	260±14
6	Blue lamp	Brake	u_6	70	S_6	330
7	Corner	Turn left	u_7	50	S_7	380±14
8	Corner	Turn right	u_8	30	S_8	410±14
9	Corner	Turn right	u_9	30	S_9	440±14
10	Corner	Turn left	u_{10}	30	S_{10}	470±14
11	Corner	Turn right	u_{11}	60	S_{11}	530±14
12	Corner	Turn right	u_{12}	30	S_{12}	560±14
13	Corner	Turn left	u_{13}	30	S_{13}	590±14
14	Corner	Turn left	u_{14}	60	S_{14}	650±14
15	Corner	Turn left	u_{15}	60	S_{15}	710±14
16	Green lamp	Brake	u_{16}	60	S_{16}	770
17	End	End	u_{17}	50	S_{17}	820

The trajectory was considered as orthogonal for simplying constraints due to the channel width of labyrinth Wc is relatively small.

Supplementary Table 5. Peak speeds of the Uni-SoPros with various characteristic length and corresponding Reynold number.

Scale	Characteristic length C_{bl} (mm)	Observed peak speed v_p (mm/s)	Re
0.4	3.4~3.6	~725.7	2467~2613
0.6	5.2~5.4	~572.9	2979~3094
0.8	6.9~7.2	~244.4	1686~1760
1.0	8.6~9.0	~244.4	2102~2200
1.2	10.3~10.8	~256.9	2646~2775
1.4	12.1~12.6	~284.5	3442~3585

Supplementary Table 6. Model parameters of the Uni-SoPro with size scale of 0.8 in deionized water.

	Sample	Parameter	Value	Parameter	Value
The Uni-SoPro with the size scale of 0.8	With fibres (n=10)	a_1	237.2	κ	0
		b_1	-18.35	T_{up} (s)	0.1
		b_2	-0.9318	T_t (s)	0.2
		b_3	-6.077	T_d (s)	0.3
		b_4	-0.8416	T_s (s)	0.3
	Without fibres (n=10)	a_1	144100	λ	-0.2019
		b_1	-0.03884	T_{up} (s)	0.05
		b_2	-3.392	T_t (s)	0.2
		b_3	-4.035	T_d (s)	0.3
		b_4	-1.359	T_s (s)	0.3

Supplementary Table 7. Nomenclatures.

	**General theory**		
$\mathbf{B}$	Magnetic field	φ	Angle between ***B*** and x-y plane
$\mathbf{B}_{x(y)(z)}$	A vector representing the x(y)(z) axis components of ***B***	h	The height of fuel tank
$\mathbf{B}_{x\text{-}y}$	A vector representing the xy plane components of ***B***	L	Length of magnetic tail
$\mathbf{B}_m$	Magnetic field during magnetization	C_{bl}	Characteristic length of Uni-SoPro
$\mathbf{M}$	Magnetization of the magnetic tail	t_c	Caliber of magnetic tail
θ	Angle between the projection of ***B*** in x-y plane and x axis	θ_d	Deflection angle of magnetic tail
d_t	The thickness of the magnetic tail		
	**Analysis for propulsion states and scaling analysis**		
F_H	Hydrostatic force	ρ_f	Density of fluid
F_V	Viscous force	δ	The thickness of boundary layer
F_T	Tension difference force	A	Amplitude of the generated wave
F_M	Momentum	S_{wet}	The wet area of the propulsor
R_W	Wave resistance	S_l	The area where convection flow acts
γ_0	Surface tension coefficient of water	l_w	Span of the wings
γ_1	Surface tension coefficient of water dipped by NOP	l_t	Span of the tail
μ_f	Dynamic viscosity of water at 20 ℃	v	Velocity of the Uni-SoPro
L_{wave}	Wavelength of the excited wave	v_c	Convection velocity
h_{water}	The depth of the experimental water	C_{wave}	The velocity of the excited water
φ_{tail}	The contact angle at the tail of Uni-SoPro	φ_{head}	The contact angle at the head of Uni-SoPro
$k_{v(h)}$	Scale factor	w_{wave}	Wave width
	**Analysis for swerving characterization**		
l_c	The axis of main body	S	Spacing of steering chips
ω_{lc}	Real time angular velocity of the l_c of main body	D	Side length of steering chips
ω	Average angular velocity of main body with SCs	ω_f	Critical parameter for describing oscillation process
	**Halt simulations**		
F_p	Propelling force	$\vec{\tau}_W$	The whole torques for tilt controlling of Uni-SoPro
	**Analysis for labyrinth constraints**		
δ_h^+	Positive error allowed in horizontal direction	Δu_h	Accumulated error in horizontal direction
δ_h^-	Negative error allowed in horizontal direction	u_i	The designed distance of step i
δ_v^+	Positive error allowed in vertical direction	S_i	The sum of designed distance of step 1~i
δ_v^-	Negative error allowed in vertical direction	W_c	The width of the tunnel
δ_i	The error of step i	W_i	The width of the lamp i
Δu_v	Accumulated error in vertical direction		
	**Analytical model for the control of the Uni-SoPros**		
θ_n	θ of step n in trajectory plan	T_{up}	The time of speeding up
l_n	Distance of step n in trajectory plan	T_t	Time of triggering
V_{fi}	The speed-time function of section i	T_s	Time of stationary steering
V_{drift}	Drift speed	T_d	Time of decelerating
κ	Correction attenuation coefficient with fibres	λ	Correction attenuation coefficient without fibres
	**Surfactant release comparisons by finite element analysis**		
c_t	The initial surfactant concentration of touching point	$\gamma(c)$	The surface tension
ε	The attenuation coefficient	γ_w	The surface tension of DI water at 20℃
c	The surfactant concentration	η	Tension coefficient associated with c
	**Mechanical analysis of the Uni-SoPros**		
E	The energy of new wave	x_c	The propulsion distance
	**Self-decoupling manipulation of the Uni-SoPros**		
v_{SC}	The volume of the steering chip	v_{mt}	The volume of the magnetic tail
$\vec{\tau}_o$	The orientation torques	$\vec{\tau}_d$	The deflection torques
	**Other**		
q	Flow rate of NOP in fuel release comparison	λ_w	Static contact angle of water
k	The laser path spacing during surface treatment	Q	The pulse width of laser cutting
v_{scan}	The speed of laser scanning	f	Pulse frequency
t	Stability time in the swerving characterizations	θ_{lc}	Included angle of body-axis and target-axis

Supplementary Methods

Detailed fabrication process of Uni-SoPros. As shown in Supplementary Fig 12, pre-prepared c-PDMS and m-PDMS precursors were spread on a polyethylene terephthalate (PET) film by an automatic coating machine (ZAA 2300, Zehntner), with the corresponding operational parameters of scraping speed of 10 mm/s, platform temperature of 45°C, and the coating thickness of 300 μm. Then, they were fully cured at 75°C for ~30 min in an oven (UF 55 plus, Memmert). Cured films were cut using a UV laser marker (HGL-LSU3/5EI, HG Laser) with a working current of 33.50 A, pulse frequency (f) of 50 kHz, a pulse width (Q) of 0.20 μs, and a scanning speed (v) of 150 mm/s for processing 7 times to obtain main bodies and SCs, where 2D patterns were designed in AutoCAD 2007 and imported into the laser operational program, EzCAD 2.12.4. The obtained main body and SCs were bonded together by PDMS precursor (weight ratio of silicone base and curing agent was 10:1) at 75°C for 20 min on a heating platform (PZ28-3TD, Harry Gestigkeit GmbH). Next, the main body assembled with SCs was laser scanned twice on both sides by the UV laser with scanning spacing (k) of 0.02 mm and a scanning speed (v_{scan}) of 800 mm/s.

The fuel tank and magnetic tail basically were soft hollow tubes, which were obtained by spin-coating or dip-coating the above c-PDMS/m-PDMS precursors onto stainless needles of different calibers (KUHNAST needle, Alibaba). The detailed processes are described as follows.

Firstly, the release agent was sprayed (Release 200, Mann) on the outer surface of a stainless needle; subsequently, the needle was dipped into prepared c-PDMS and m-PDMS, and then cured to make tanks and tails, respectively. In the case of smaller propulsors with a size scale less than 0.8 (include 0.8), after dipped with prepared uncured silicone mixture, the needle would be mounted on a rotary tool (Stylo+ 2050, Dremel) and spun at a specific speed for 6 s, otherwise, the needle with prepared material would be cured at 75°C for ~30 min directly in an oven (UF 55 plus, Memmert). Upon crosslinked, hollow c-PDMS/m-PDMS tubes were taken out and cleaned by spraying with IPA (Shenshi Chemical Industry Corporation) for the next step of processing.

Secondly, obtained tubes were trimmed and cut to the appropriate length with matched connecting holes, using the laser with the v_{scan} of 150 mm/s and the Q of 0.20 μs for processing 3 times to obtain fuel tanks and magnetic tails.

Thirdly, the obtained fuel tank and magnetic tail were assembled together through silica gel (Sil-Poxy, Smooth-On) gluing under a microscope (SZM0745-STL1-T1, Chenyu Optoelectronics Technology Co., Ltd.), and finally, were attached to the main body with steering chips via silica gel gluing to form an insect-scale soft propulsor.

Superhydrophobic surface treatments. The same UV laser was used to tune c-PDMS/m-PDMS surface morphology for a more hydrophobic surface. The detailed sample preparation and characterization were as follows: c-PDMS and m-PDMS precursors mixtures were spread on a glass slide (REF.10127101P-G, Citotest) by a 500 μm applicator (SZQ-830, Alibaba) and cured at 75°C for 30 min on a heating platform (PZ28-3TD, Harry Gestigkeit GmbH). After curing, the sample surfaces were then modified by the laser with different operational parameters of scanning spacing k (from 0.02 mm to 0.16 mm) and the scanning speed v_{scan} (from 100 to 800 mm/s). The detailed surface morphologies were obtained, using a scanning electron microscope (SU3900, HITACHI). Each static contact angle of c-PDMS and m-PDMS with different k and v, was measured using a drop analyzer (DSA25, Kruss) via a sessile drop method.

Magnetic characterizations. The magnetic moment densities of the NdFeB microparticles and m-PDMS film specimens were measured by a vibrating sample magnetometer (S-VSM, Quantum). The specimens were prepared by weighing ~4 mg of NdFeB powder via an electronic balance (SQP, Sartorius) and cutting m-PDMS film with various NdFeB mass fractions from 15% to 75% into circles (diameter of 6 mm, thickness of ~0.1 mm) through the laser. We measured the residual magnetization of NdFeB powder and m-PDMS film (mass fraction of 65.57%) under applied magnetic fields from 5,000 Oe to 50,000 Oe, respectively, Supplementary Fig. 6a, b. In addition, we set the applied magnetic field to 24,000 Oe and measured the magnetization of m-PDMS film with a mass fraction from 15% to 75%. The field change rate was set to 500 Oe/s for film specimens and 700 Oe/s for magnetic powder specimens, respectively. The temperature in the cavity was set to 300 K for all measurements.

Decoupled control. The 3D magnetic field **B** can be orthogonally decomposed into a horizontal component $\mathbf{B}_{x\text{-}y}$ and a vertical component $\mathbf{B}_z$. Based on the presented design, we utilized $\mathbf{B}_{x\text{-}y}$ to achieve swerving control and $\mathbf{B}_z$ to trigger the deflection of the magnetic tail to tune propulsion

behavior, and hence overall control the movement of Uni-SoPros. Via this strategy, only one 3D magnetic field **B** can simultaneously and precisely control the propulsion and swerving.

Fluidic delivery with/without fibres. To explore the fibre's influence on the delivery and release behaviors of surfactant (NOP), a series of comparative experiments have been conducted. For the quasi-process of NOP release, the magnetic tails with/without inserted fibres were vertically placed (Supplementary Fig 9a). A syringe pump (Harvard Apparatus, Pump 11 Elite) was connected to dispense surfactant out with a flow of 5 μl/min. The whole dispensing process was recorded by the drop-shape analyzer (DSA25, Kruss). For spreading visualization, the surfactant stored in the fuel tank was colored green by diesel colorant. Using the magnetic field to deflect the magnetic tail and make it touch the water surface for ~100 ms, the SLR camera was used to record the distribution of NOP on water surface (85 mm in diameter, ~7 mm in depth, Supplementary Fig 9c). The dynamic process of NOP spreading on the water (85 mm in diameter, ~5 mm in depth) with/without fibres was recorded using the high-speed camera, where the specimen was a magnetic tail connected to a fuel tank (the size scale was 1.4). All experiments were conducted with deionized water (20°C).

Cross through the labyrinth with dynamic lights. A Uni-SoPro with a size scale of 0.6 was used in this demo. The propulsor crossed through a 7 by 10 orthogonal labyrinth that was customized made of 3D printed resin (8000, Wenext). The dynamic signal lights were adhesively attached to the predesigned positions of the printed labyrinth. During the demo, the labyrinth was filled with deionized water of ~ 1cm and placed in the center of the Helmholtz coil. The video was recorded by the SLR camera. The running rules were defined as follows: the Uni-SoPro should cross through the labyrinth as quickly as possible; the Uni-SoPro should stop immediately when the frontal LED lights up, and pass by as soon as possible when the LED lights off; the Uni-SoPro should not strike any walls in the whole process. The critical parameters of the dynamic labyrinth are in Supplementary Table 4.

Dodging a predator. A Uni-SoPro with a size scale of 0.4 was used in this demo. The scene was built in an acrylic container (20 cm×20 cm×5 cm) and placed in the Helmholtz coil. A pneumatic elongate blower (95W6E, Alibaba) was installed into a toy lizard to mimic the tongue of a predator. It was curled in the initial state and can be elongated quickly (length of ~7 cm and width of ~8

mm) in ~40 ms by an air actuation under the pressure of ~0.3 MPa. Using the high-speed camera above, the interaction of the predator (toy lizard) and prey (Uni-SoPro) was observed. Simultaneously, the SLR camera on the oblique view was used to record the scene complimentary (Supplementary Movie 3).

Supplementary References

1. T. Xu, J. Yu, X. Yan, H. Choi, L. Zhang, Magnetic actuation based motion control for microrobots: An overview. *Micromachines*. **6**, 1346–1364 (2015).
2. J. W. M. Bush, D. L. Hu, Walking on water: Biolocomotion at the interface. *Annu. Rev. Fluid Mech.* **38**, 339–369 (2006).
3. T. R. Akylas, C. C. Mei, Wave resistance of a two-dimensional obstacle, 1–14.
4. N. D. Katopodes, in *Free-Surface Flow* (Elsevier, 2019; https://linkinghub.elsevier.com/retrieve/pii/B9780128154892000095), pp. 652–708.
5. D. K. P. Yue, Marine hydrodynamics OpenCourse, Lecture notes Chapter 6 - Water Waves. *Http://Ocw.Mit.Edu/Courses/Mechanical-Engineering/2-20-Marine-Hydrodynamics-13-021-Spring-2005/Lecture-Notes/*, 1–14 (2005).
6. Z. H. Zhu, D. Yang, M. J. Rosen, Some synergistic properties of N- Alkyl- 2- pyrrolidones, a New class of surfactants 1. *J. Am. Oil Chem. Soc.* **66**, 998–1001 (1989).
7. MIT open course, Under, Over and Critical Damping 1. Response to Damping, 2–7 (2011).
8. G. Amendola, M. Fabrizio, J. M. Golden, Exponential Decay. *Thermodyn. Mater. with Mem.*, 459–474 (2012).
9. B. Wang, S. Handschuh-Wang, J. Shen, X. Zhou, Z. Guo, W. Liu, M. Pumera, L. Zhang, Small-Scale Robotics with Tailored Wettability. *Adv. Mater.* **35**, 1–28 (2023).
10. L. X. Lyu, F. Li, K. Wu, P. Deng, S. H. Jeong, Z. Wu, H. Ding, Bio-inspired untethered fully soft robots in liquid actuated by induced energy gradients. *Natl. Sci. Rev.* **6**, 970–981 (2019).
11. A. M. Wilson, T. Y. Hubel, S. D. Wilshin, J. C. Lowe, M. Lorenc, O. P. Dewhirst, H. L. A. Bartlam-Brooks, R. Diack, E. Bennitt, K. A. Golabek, Biomechanics of predator–prey arms race in lion, zebra, cheetah and impala. *Nature*. **554**, 183–188 (2018).
12. R. M. Walter, Kinematics of 90 running turns in wild mice. *J. Exp. Biol.* **206**, 1739–1749 (2003).
13. M. Badura, Biomechanical analysis of the discus at the 2009 IAAF World Championships in athletics. *New Stud. Athl.* **25**, 23–35 (2010).
14. S. A. Combes, D. E. Rundle, J. M. Iwasaki, J. D. Crall, Linking biomechanics and ecology through predator–prey interactions: flight performance of dragonflies and their prey. *J. Exp. Biol.* **215**, 903–913 (2012).
15. Y. Zeng, S. Crews, Biomechanics of omnidirectional strikes in flat spiders. *J. Exp. Biol.* **221**, jeb166512 (2018).
16. A. Koehnsen, J. Kambach, S. Büsse, Step by step and frame by frame – Workflow for efficient motion tracking of high-speed movements in animals. *Zoology*. **141**, 125800 (2020).
17. F. T. Muijres, S. W. Chang, W. G. van Veen, J. Spitzen, B. T. Biemans, M. A. R. Koehl, R. Dudley, Escaping blood-fed malaria mosquitoes minimize tactile detection without compromising on take-off speed. *J. Exp. Biol.* **220**, 3751–3762 (2017).
18. V. M. Ortega-Jimenez, L. Von Rabenau, R. Dudley, Escape jumping by three age-classes of water striders

from smooth, wavy & bubbling water surfaces. *J. Exp. Biol.* **220**, 2809–2815 (2017).

19. P. W. WEBB, Speed, Acceleration and Manoeuvrability of Two Teleost Fishes. *J. Exp. Biol.* **102**, 115–122 (1983).
20. R. C. Eaton, R. A. Bombardieri, D. L. Meyer, The Mauthner-initiated startle response in teleost fish. *J. Exp. Biol.* **66**, 65–81 (1977).
21. H.-T. Lin, G. G. Leisk, B. Trimmer, GoQBot: a caterpillar-inspired soft-bodied rolling robot. *Bioinspir. Biomim.* **6**, 026007 (2011).
22. A. D. Marchese, C. D. Onal, D. Rus, Autonomous soft robotic fish capable of escape maneuvers using fluidic elastomer actuators. *Soft Robot.* **1**, 75–87 (2014).
23. H. Peng, T. Mao, X. Lu, A small legged deformable robot with multi-mode motion. *J. Intell. Mater. Syst. Struct.* **31**, 704–718 (2020).
24. M. T. Tolley, R. F. Shepherd, M. Karpelson, N. W. Bartlett, K. C. Galloway, M. Wehner, R. Nunes, G. M. Whitesides, R. J. Wood, in *2014 IEEE/RSJ International Conference on Intelligent Robots and Systems* (2014), pp. 561–566.
25. Sangok Seok, C. D. Onal, R. Wood, D. Rus, Sangbae Kim, in *2010 IEEE International Conference on Robotics and Automation* (IEEE, 2010; http://ieeexplore.ieee.org/document/5509542/), pp. 1228–1233.
26. D. Zarrouk, R. S. Fearing, Controlled in-plane locomotion of a hexapod using a single actuator. *IEEE Trans. Robot.* **31**, 157–167 (2015).
27. D. W. Haldane, R. S. Fearing, in *2015 IEEE International Conference on Robotics and Automation (ICRA)* (IEEE, 2015; http://ieeexplore.ieee.org/document/7139828/), vols. 2015-June, pp. 4539–4546.
28. J. G. Cham, S. A. Bailey, J. E. Clark, R. J. Full, M. R. Cutkosky, Fast and robust: Hexapedal robots via shape deposition manufacturing. *Int. J. Rob. Res.* **21**, 869–882 (2002).
29. D. W. Haldane, R. S. Fearing, in *Robotics: Science and Systems* (The MIT Press, 2008; https://direct.mit.edu/books/book/2310/chapter/60363/design-of-a-bio-inspired-dynamical-vertical), pp. 9–16.
30. J.-S. Koh, E. Yang, G.-P. Jung, S.-P. Jung, J. H. Son, S.-I. Lee, P. G. Jablonski, R. J. Wood, H.-Y. Kim, K.-J. Cho, Jumping on water: Surface tension–dominated jumping of water striders and robotic insects. *Science.* **349**, 517–521 (2015).
31. M. Ilton, M. Saad Bhamla, X. Ma, S. M. Cox, L. L. Fitchett, Y. Kim, J. sung Koh, D. Krishnamurthy, C. Y. Kuo, F. Z. Temel, A. J. Crosby, M. Prakash, G. P. Sutton, R. J. Wood, E. Azizi, S. Bergbreiter, S. N. Patek, The principles of cascading power limits in small, fast biological and engineered systems. *Science*. **360** (2018), doi:10.1126/science.aao1082.
