# Supplementary material for: Stenus-inspired, swift, and agile untethered insect-scale soft propulsors
Source: Nat Commun. 2024 Feb 19;15:1491. doi: 10.1038/s41467-024-45997-3 (PMC10876683; doi:10.1038/s41467-024-45997-3)
Supplement: Supplementary file 3 — Description of Additional Supplementary Files [file 41467_2024_45997_MOESM3_ESM.pdf]

Description of Additional Supplementary Files

Supplementary Movie Legends

Supplementary Movie 1. Swift moving of the untethered insect-scale soft propulsor

Supplementary Movie 2. Agile braking of the untethered insect-scale soft propulsor

Supplementary Movie 3. Swift and Agile motion of the untethered insect-scale soft propulsor

Supplementary Movie 4. Decoupled steering and magnetic tail control

Supplementary Movie 5. Computation fluid dynamic analysis of the braking process of the Uni-SoPro

Supplementary Movie 6. The soft propulsor escapes rapidly from violent predation of a lizard

Supplementary Movie 7. Passing through a labyrinth with dynamic signal lamps

Supplementary Movie 8. Introduction of the untethered insect-scale soft propulsor
